# Taxonomer: an interactive metagenomics analysis portal for universal pathogen detection and host mRNA expression profiling

**DOI:** 10.1186/s13059-016-0969-1

**Published:** 2016-05-26

**Authors:** Steven Flygare, Keith Simmon, Chase Miller, Yi Qiao, Brett Kennedy, Tonya Di Sera, Erin H. Graf, Keith D. Tardif, Aurélie Kapusta, Shawn Rynearson, Chris Stockmann, Krista Queen, Suxiang Tong, Karl V. Voelkerding, Anne Blaschke, Carrie L. Byington, Seema Jain, Andrew Pavia, Krow Ampofo, Karen Eilbeck, Gabor Marth, Mark Yandell, Robert Schlaberg

**Affiliations:** Department of Human Genetics, University of Utah, Salt Lake City, UT USA; Department of Biomedical Informatics, University of Utah, Salt Lake City, UT USA; Department of Pathology, University of Utah, Salt Lake City, UT USA; ARUP Institute for Clinical and Experimental Pathology, Salt Lake City, UT USA; Department of Pediatrics, University of Utah, Salt Lake City, UT USA; Centers for Disease Control and Prevention, Atlanta, GA USA; USTAR Center for Genetic Discovery, Salt Lake City, UT USA

**Keywords:** Metagenomics, Microbiome, Pathogen detection, Infectious disease diagnostics

## Abstract

**Background:**

High-throughput sequencing enables unbiased profiling of microbial communities, universal pathogen detection, and host response to infectious diseases. However, computation times and algorithmic inaccuracies have hindered adoption.

**Results:**

We present Taxonomer, an ultrafast, web-tool for comprehensive metagenomics data analysis and interactive results visualization. Taxonomer is unique in providing integrated nucleotide and protein-based classification and simultaneous host messenger RNA (mRNA) transcript profiling. Using real-world case-studies, we show that Taxonomer detects previously unrecognized infections and reveals antiviral host mRNA expression profiles. To facilitate data-sharing across geographic distances in outbreak settings, Taxonomer is publicly available through a web-based user interface.

**Conclusions:**

Taxonomer enables rapid, accurate, and interactive analyses of metagenomics data on personal computers and mobile devices.

**Electronic supplementary material:**

The online version of this article (doi:10.1186/s13059-016-0969-1) contains supplementary material, which is available to authorized users.

## Background

Metagenomics, the genomic analysis of a population of microorganisms, makes possible the profiling of microbial communities in the environment and the human body at unprecedented depth and breadth. Its rapidly expanding use is revolutionizing our understanding of microbial diversity in natural and man-made environments and is linking microbial community profiles with health and disease [1–9]. To date, most studies have relied on PCR amplification of microbial marker genes (e.g. bacterial 16S ribosomal RNA [rRNA]), for which large, curated databases have been established [10–12]. More recently, higher throughput and lower cost sequencing technologies have enabled a shift towards enrichment-independent metagenomics. These approaches reduce bias, improve detection of less abundant taxa, and enable discovery of novel pathogens [13–15]. In addition, they promise to revolutionize how infectious diseases are diagnosed.

With replacement of microbial culture by molecular tests, the laboratory diagnosis of infectious diseases increasingly relies on pathogen-specific tests. While more sensitive, they require *a priori* knowledge of likely etiologic agents (i.e. answering the question “is pathogen X present”). For several common syndromes (e.g. pneumonia, sepsis, encephalitis), many different pathogens can cause clinically indistinguishable symptoms. Thus, increasingly large yet inherently limited diagnostic panels are necessary for detection of common pathogens and extensive follow-up testing may be required if first-line tests are negative. In contrast, enrichment-independent next-generation sequencing (NGS) allows for unbiased, hypothesis-free detection and molecular typing of a theoretically unlimited number of common and unusual pathogens (i.e. answering the question “what pathogen is present”). Unbiased, NGS-based pathogen detection has led to the diagnosis of previously unrecognized infections and discovery of novel pathogens in select cases (see [16] for example). A unified approach for detection of potential pathogens will increase diagnostic yield, decrease time to result for unexpected pathogens, improve targeted treatment, and will aid in the rapid response to public health emergencies.

While direct pathogen identification from sequencing data is generally the goal, even when a specific causative pathogen cannot be identified, differentiating viral from bacterial infections, for example, can indicate whether antibiotic treatment is necessary. In the past, this has been attempted through assessment of the leukocyte response, protein markers (e.g. procalcitonin), or microarray-based host transcript expression profiling from blood leukocytes [17–19]. The greater sensitivity and unbiased nature of RNA sequencing (RNA-seq) enables simultaneous pathogen detection and host-expression response profiling, which in theory could be used to better inform treatment, potentially overcoming many of the limitations of current approaches [20, 21], even in the absence of a definitive diagnosis of a pathogen.

NGS also enables more comprehensive microbial profiling studies. For example, dysbiosis of the mucosal and cutaneous microbiota has been linked to metabolic, immunologic, cardiovascular, and neoplastic diseases [5, 22–26]. However, today most microbiome studies still rely upon PCR amplification of marker genes (e.g. bacterial 16S rRNA). This approach introduces bias [13], ignores effects of the relevant viral and phage flora for which no marker gene exists [27–29], and is unable to assess host response differences, all of which are known to influence the outcome of infectious diseases and modulate human microbial communities.

Wide availability of NGS instruments, lower reagent costs, and streamlined sample preparation protocols have enabled an increasing number of investigators to perform high-throughput DNA and RNA-seq for metagenomics studies. Unfortunately, analysis of the large datasets generated by high-throughput metagenomics requires a combination of bioinformatics skills, computational resources, and microbiological expertise that is absent from most laboratories, especially diagnostic ones. Thus, more computationally efficient, accurate, and easy-to-use tools for comprehensive diagnostic and metagenomics analyses are needed.

## Results

### Description of taxonomer

**Fig. 1:**
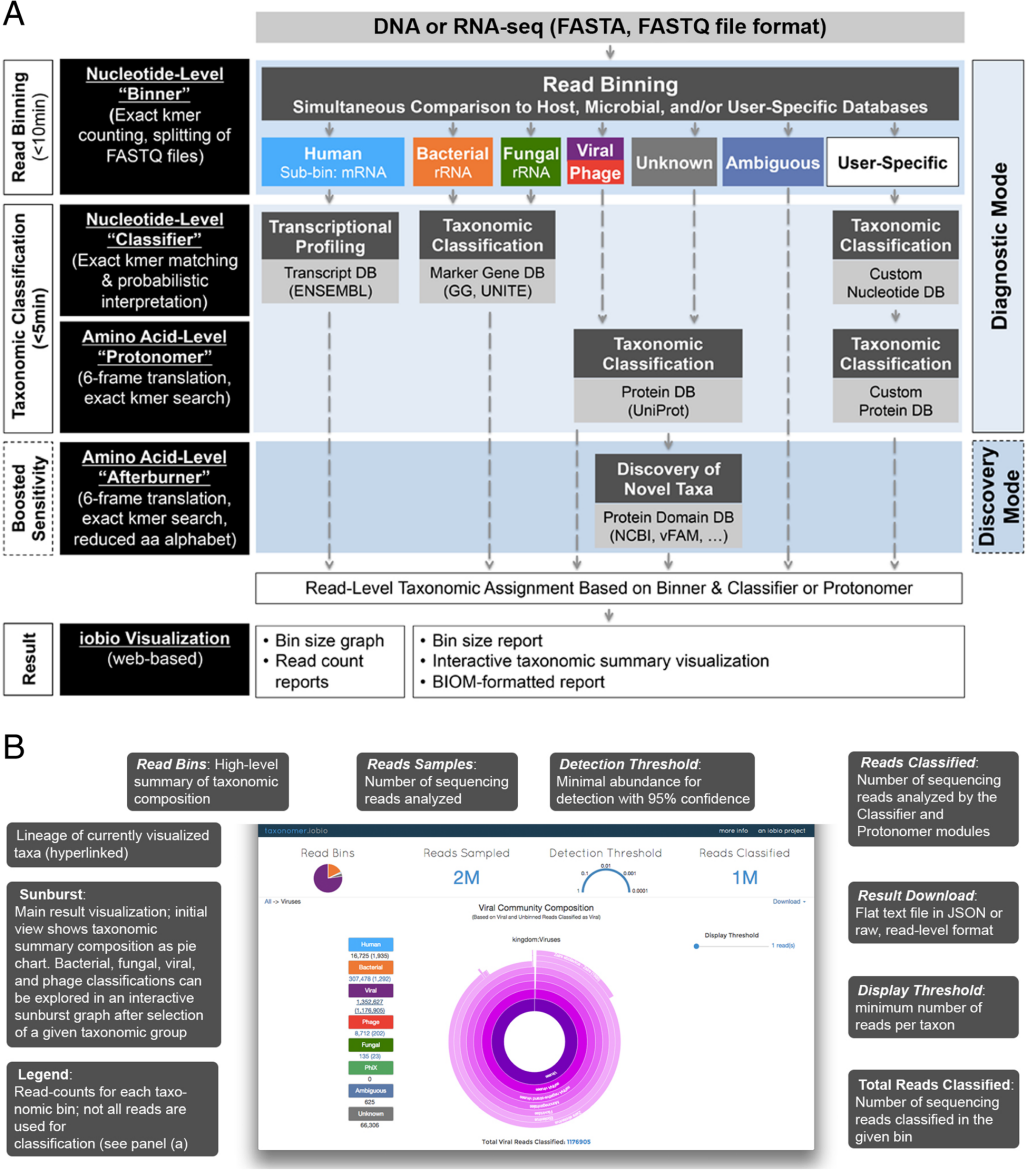
Overview of Taxonomer architecture and user interface. **a** Taxonomer’s architecture. Raw FASTA, FASTQ, or SRA files (with or without gzip compression) are the input for Taxonomer. For paired-end data, mate pairs are analyzed jointly. Taxonomer consists of four main modules. The “Binner” module categorizes (“bins”) reads into broad taxonomic groups (host and microbial) followed by comprehensive microbial and host gene expression profiling at the nucleotide (“Classifier” module) or amino acid-level (“Protonomer” and “Afterburner” modules). Normalized host gene expression (gene-level read counts) and microbial profiles can be downloaded. Read subsets can be downloaded for custom downstream analyses (**b**) Taxonomer web-service. To further remove barriers for academic and clinical adoption of metagenomics, we developed a web interface for Taxonomer that allows users to stream sequencing read files (stored locally or http accessible) to the analysis server and interactively visualize results in real time. Main features are described in *grey boxes*. Taxonomic classification of bacteria, fungi, and viruses is visualized as a *sunburst graph* (*center*), in which the size of a given slice represents the relative abundance at the read level. Taxonomic ranks are shown hierarchically with the highest rank in the *center of the graph*. Sequences that cannot be classified to the species level, either because they are shared between taxa or represent novel microorganisms, are collapsed to the lowest common ancestor and shown as part of slices that terminate at higher taxonomic ranks (e.g. genus, family)

Taxonomer is an ultrafast, user-friendly, web-based metagenomic sequence analysis tool. It enables novel analysis modalities in an easy-to-use fashion including: (1) comprehensive panmicrobial detection and discovery; (2) host messenger RNA (mRNA) response profiling; (3) interactive result visualization; and (4) access through a web-based user interface, which eliminates the need for specialized hardware or expertise. These applications are enabled through a modular design based on four integrated tools: Binner, Classifier, Protonomer, and Afterburner (Fig. 1a). Taxonomer can be used in the analysis of DNA and/or RNA sequencing data as well as for short reads and longer contigs assembled from metagenomics datasets. Taxonomer operates at speeds comparable to the fastest tools, Kraken [30] (~4 million reads/min) and CLARK [31] (~32 million reads/min), which provide only some of Taxonomer’s functionality. Unlike Kraken and CLARK, Taxonomer supports integrated nucleotide and protein-based classification for detection of highly diverse viral sequences at 10–100 times faster speeds than alignment-based tools with similar functionality (e.g. those used by SURPI [32]).

### Binner module

Rapidly identifying small numbers of pathogen sequences hidden among vast numbers of host and/or microbiota-derived sequencing reads is a major algorithmic challenge for metagenomics-based pathogen detection tools. The conventional approach is to use digital subtraction of host sequences [64], whereby all sequencing reads are first aligned to the host’s genome sequence. This is the approach used by SURPI, for example. Additional subtraction steps may be used for removal of non-relevant microbial sequences, including those known to represent reagent contamination (e.g. [43, 62]). A greatly reduced number of presumably relevant microbial sequences are then classified by computationally intense alignment to larger reference databases. Since only the remaining reads are matched with microbial reference sequences, pathogens can be missed entirely if they are homologous to sequences in the subtraction database. Taxonomer overcomes this inherent limitation of digital subtraction by means of its “Binner” module (Fig. 1a, Additional file 1: Figure S1), which compares each read to every reference database in parallel, assigning them to broad, non-exclusive taxonomic categories.

### Classifier module

Nucleotide-level classification in Taxonomer is based on exact k-mer matching. Taxonomer uses databases that are optimized for rapid k-mer queries that store every reference in which a k-mer is found as well as an associated k-mer weight for every reference. Each read is assigned to the reference that has the maximum total k-mer weight. In the case of a tie, the query sequence is assigned to the taxonomic lowest common ancestor (LCA). The classifier module is used for rRNA-based bacterial and fungal characterization and host mRNA expression profiling.

### Protonomer module

Taxonomer uses a novel a non-degenerate mapping scheme between amino acids and corresponding, artificial DNA sequences to facilitate mapping in protein space with the same algorithm used for classification in nucleotide space. Query reads are translated into all six reading frames based on the same non-degenerate translation scheme and classified in all frames. K-mer weighting and read classification assignment are performed as with the Classifier module. Protonomer is used to classify viruses in protein space because of their high mutation rates, genetic variability, and incomplete reference databases [58].

### Afterburner

To increase recovery of distantly homologous viral proteins, Taxonomer offers two options. First, unclassified reads can be further analyzed using the Afterburner module, a degenerate k-mer matching engine that employs a collapsed amino-acid alphabet. In a manner similar to that employed by DIAMOND [37], we used k-means clustering on the BLOSUM62 matrix to generate a compressed amino acid alphabet. By using the collapsed amino acid alphabet, Taxonomer achieves higher sensitivity in classification with sequences that are more diverged at the expense of a higher false-positive rate when compared with Protonomer.

### Databases

Bacterial classification is based on a marker gene approach (16S rRNA gene) and the Greengenes database [45, 70]. Fungal classification is also based on a marker gene approach (internal transcribed spacer, ITS, rRNA sequences) using the UNITE database [60]. For viral classification and discovery, Taxonomer uses the viral subset of UniRef90 [71] combined with the bacterial subset of UniRef50. Human mRNA transcript expression profiling is based on transcripts and corresponding gene models from the ENSMBL human reference sequence.

Taxonomer is available via an intuitive iobio [33] web-service (Fig. 1b), allowing rapid, highly interactive analyses accessible through personal computers and mobile devices without the need for special computational infrastructure on the user side.

## Processing time and completeness

To demonstrate the power and utility of Taxonomer, we carried out benchmark analyses using biological and synthetic datasets. These include a large number of pediatric nasopharyngeal (NP)/oropharyngeal (OP) swabs from the Centers for Disease Control and Prevention (CDC) Etiology of Pneumonia In the Community (EPIC) study [40] as well as published data [41–43]. We also compared Taxonomer’s speed and classification accuracy to state-of-the-art tools for sequence alignment (BLAST [34]), rapid metagenomic data analysis (Kraken, SURPI), marker gene-based microbial classification (RDP Classifier [35]), protein searches (RapSearch2 [36], DIAMOND [37]), and RNA-seq-based transcriptional profiling (Sailfish [38] and Cufflinks [39]).

### Speed and completeness of classification

We used RNA-seq data from three virus-positive NP/OP samples with a range of host versus microbial composition profiles to compare speed and completeness of classification by Taxonomer, to two other ultra-fast metagenomics tools: Kraken and SURPI (Table 1). Respiratory viruses were confirmed by routine methods [40, 44]. Kraken was the fastest tool (mean 1.5 min/sample), but classified the fewest reads because it relies on nucleic acid-level classification alone and uses a single reference database. Although SURPI enables amino acid-level searches for virus detection and discovery, this greatly extended analysis times to between 1.5 and >12 h/sample. Taxonomer achieved run times similar to Kraken (~5 min/sample, 5–8 × 10^6^ reads/sample), while performing nucleotide and protein-based microbial classification as well as host gene expression profiling. Taxonomer also classified the largest number of reads. Collectively these results demonstrate how Taxonomer combines the ultrafast speed of Kraken with an extended suite of analysis and search capabilities that exceed those of SURPI.

**Table 1:** Processing time of Taxonomer compared to rapid classification pipelines SURPI and Kraken. RNA-Seq data generated from three nasopharyngeal specimens with varying taxonomic composition illustrate differences in analysis times between the three tools. (Human: blue; Bacteria: orange; Fungal: green; Virus: red; Other: yellow; Unclassified: gray)

Sample composition, total reads	Pathogen	Application	Subtraction	Binning	Classification	Protein search	Total time	% Reads classified
6,599,164	HCoV	Taxonomer	-	5 min	22 s	10 s	5.5 min	99.9 %
		Kraken	-	-	1.5 min	-	1.5 min	99.6 %
		SURPI	3.3 min	-	74 min	15 min	92 min	99.9 %
7,542,552	Influenza A virus	Taxonomer	-	8 min	40 s	30 s	9.2 min	88 %
		Kraken	-	-	1.5 min	-	1.5 min	66 %
		SURPI	9.8 min	-	208 min	18 min	236 min	78 %
6,252,311	HMPV	Taxonomer	-	5.2 min	56 s	10 s	6.3 min	98 %
		Kraken	-	-	1.3 min	-	1.3 min	93 %
		SURPI	56 min	-	648 min	24 min	728 min	95 %

## Read binning

To demonstrate the advantage of Taxonomer’s non-greedy binning algorithm, we compared high-level taxonomic assignments made by SURPI, which employs greedy digital subtraction using sequence alignments by SNAP [67], to those of Taxonomer’s alignment-free Binner (Additional file 1: Figure S2). While high-level taxonomic assignments agree for 73.8 % of RNA-seq reads, Taxonomer assigned 16 % of reads an ambiguous origin (i.e. they match equally to multiple databases), 96 % of these were classified as human by SURPI. This was mostly due to highly conserved ribosomal and mitochondrial sequences (data not shown), but similar effects were also apparent for fungal sequences (18 % classified as human by SURPI). Taxonomer’s Binner was also able to capture more phage/viral sequences (7426) than the alignment-based method (5798), and resulted in fewer unclassified sequencing reads (3.2 % vs. 4.5 %) (Additional file 1: Table S1). Consistent with lower abundance of rRNA and mitochondrial RNA (mtRNA) sequences in DNA-sequencing data, Taxonomer had many fewer ambiguous assignments (0.04 %, of which 40 % were classified as human and 59 % as viral by SURPI; overall agreement 98.7 %).

## Bacterial and fungal classification

### Bacterial and fungal classification

Reads derived from taxa that are absent from classification databases can result in false-negative and false-positive classifications, especially at the genus and species level (Additional file 1: Figure S3). Thus, comprehensive classification databases are essential and several options exist. RefSeq contains whole genome sequences of only ~5000 bacterial taxa (www.ncbi.nlm.nih.gov/refseq/), whereas more comprehensive 16S rRNA sequence databases [12, 35, 45] suggest existence of 100,000–200,000 species. As a result, 16S reads from unrepresented bacteria are more readily identified than reads derived from other genomic targets (Additional file 1: Figure S2). To maximize classification accuracy, Taxonomer employs a 16S marker gene approach and a custom Greengenes-derived database.

### Default benchmarks

**Fig. 2:**
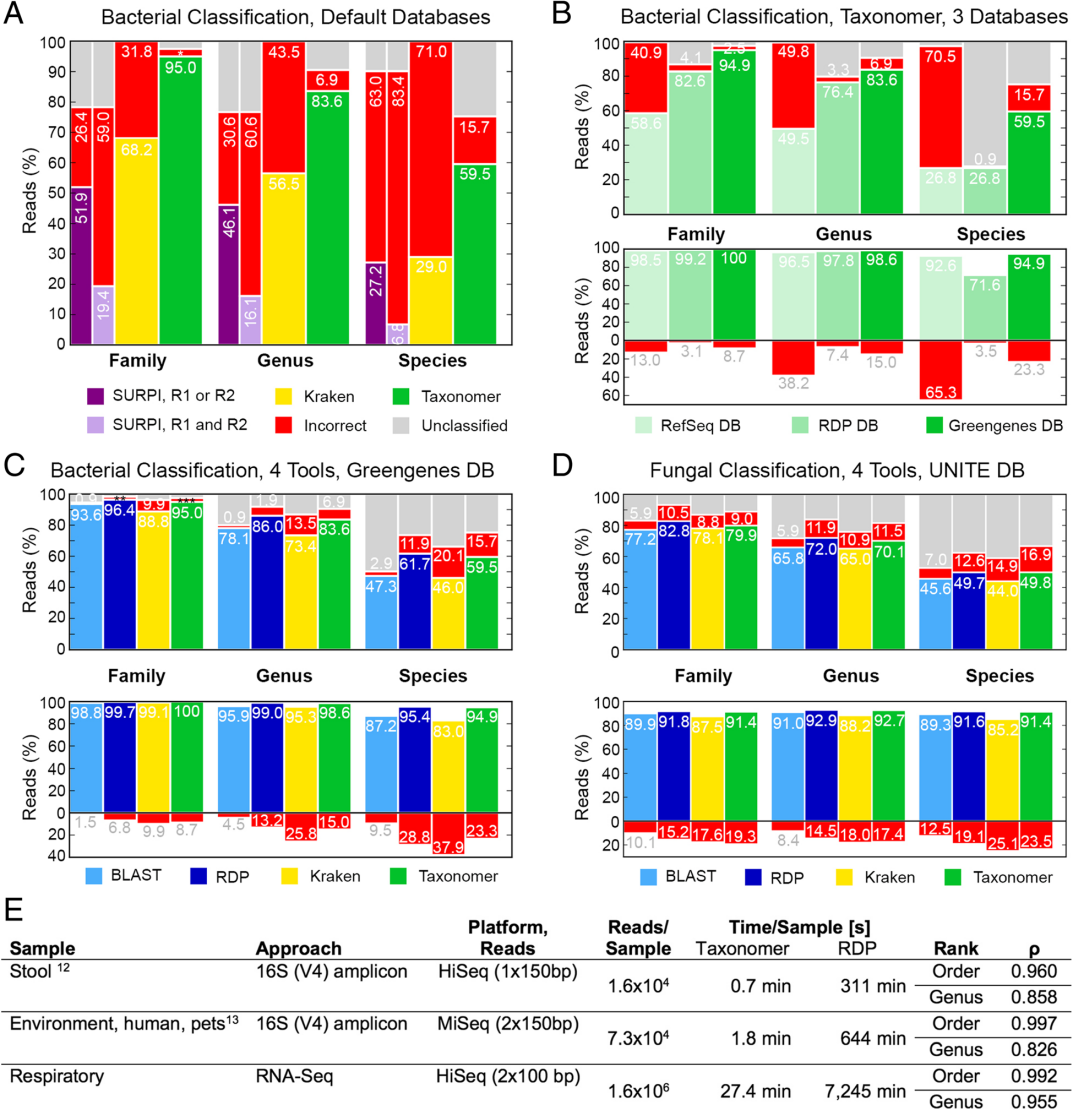
Performance of the “Classifier” module for bacterial and fungal classification and bacterial community profiling. **a** Taxonomer provides superior sensitivity and specificity for read-level bacterial classification compared to two other rapid classification tools SURPI [32] and Kraken [30] when using each tool’s default settings and databases: nt (www.ncbi.nlm.nih.gov/nucleotide, SURPI), RefSeq (Kraken), and Greengenes 99 % [70] OTU (Taxonomer). Results for SURPI are based on correct identification by either (*dark bar*) or both (*light bar*) read mates. **b** Of the three commonly used reference databases RefSeq (*n* = 210,627; 5,242 bacterial genomes), Greengenes 99 % OTU (*n* = 203,452), and RDP (*n* = 2,929,433), Taxonomer provides greatest read-level (*top*) and taxon-level (*bottom*, i.e. percentage of bacterial species identified) sensitivity for bacterial classification at only a moderate decrease in specificity when using the Greengenes database compared to the RDP and RefSeq databases (simulated 16S rDNA as in **a**). Because of its large size and greater completeness, the RDP database provides the greatest species-level specificity at the tradeoff of sensitivity. For ease of reference, the *top right-most column* is repeated from (**a**). **c** Bacterial classification accuracy of Taxonomer is similar to the RDP Classifier [35] and superior to Kraken at the read-level (*top*) and taxon-level (*bottom*, all using the Greengenes database). Given the applied criteria, BLAST [34] is less sensitive but more specific. **d** Taxonomer also performs similar to the RDP Classifier and better than Kraken for classification of synthetic fungal internal transcribed spacer (ITS) sequences at the read-level (*top*) and taxon-level (*bottom*). **e** Taxonomer classifies bacterial 16S rRNA reads at >200-fold increased speed compared to the RDP Classifier (times for 1 CPU, multithreading not available for RDP Classifier) while providing highly comparable bacterial community profiles when using 16S rRNA gene amplicon sequencing and shotgun metagenomics. Spearman correlation coefficients (ρ) of abundance estimates are shown for Taxonomer and the RDP Classifier at the order and genus-levels using the Greengenes 99 % OTU reference database. *2.5 %; **1.9 %; ***2.5 %

Performance of classification tools is frequently only tested with synthetic reads derived from the reference database; i.e. perfect matches exist for all synthetic reads. This is a highly artificial challenge, as novel microbial species or strains are routinely encountered in clinical or environmental samples for which perfect matches do not exist in the reference database. To provide a more realistic challenge, we generated synthetic reads from phylogenetically diverse 16S sequences [12] almost half (n = 468, 46 %) of which lacked perfect matches in Taxonomer’s reference database (Additional file 1: Table S2). The utility of Taxonomer’s k-mer weighting approach (see “Methods”) is illustrated in Fig. 2a, demonstrating superior accuracy compared to SURPI and Kraken when using each tool’s default databases and command lines. At the species level, Taxonomer correctly classified 59.5 %, incorrectly classified 15.7 %, and failed to classify 24.8 % of the reads. By comparison, Kraken classified 29 % of the reads to the correct species but classified every remaining read (71 %) incorrectly. As SURPI aligns each read from a mate pair independently and in many cases best matches are discordant (Additional file 1: Table S3), results are shown for correct classification of either (left half) or both read mates (right half). In both analyses, SURPI underperformed Taxonomer and Kraken.

### Database benchmarks

Next, we assessed the effect of three different databases (RefSeq, RDP [35], and Taxonomer’s custom Greengenes-derived database) on Taxonomer’s accuracy using the same synthetic reads (Fig. 2b). With the Greengenes-derived database, Taxonomer correctly classified 59.5 % of the reads at the species level and recovered 94.9 % of species. Using RefSeq (Kraken’s default database), Taxonomer’s values drop to 27 % and 71.6 %, respectively, similar to Kraken’s results when using the same database: 29 % and 71 %, respectively. Although Taxonomer misclassified very few reads using the RDP database, overall performance was inferior. Thus, Taxonomer’s Greengenes-derived database is its default for bacterial classification.

### Algorithmic benchmarks

To compare accuracy of classification algorithms, we used the same database (Taxonomer’s Greengenes-derived db), and classified the same synthetic reads with Taxonomer, MegaBLAST (www.ncbi.nlm.nih.gov/blast/html/megablast.html), RDP Classifier [35], and Kraken (Fig. 2c). SURPI was not included, as it provides no means to replace its reference databases. Overall, Taxonomer’s performance closely approximated that of the RDP Classifier, an established reference tool (59.5 % and 61.4 % correct species-level classifications, respectively). Kraken’s performance improved using the Taxonomer’s Greengenes-derived database, but Taxonomer still correctly classified 13.5 % more reads, had a lower false-positive rate (15.7 % vs. 20.1 %), recovered more taxa correctly (94.9 % vs. 83 %), and had a lower false recovery rate (23.3 % vs. 37.9 %). Similar performance advantages are also seen for fungal classification and recovery rates using Taxonomer’s ITS database (Fig. 2d). Lastly, we examined the impact of read length, sequencing error rates, and Kraken’s confidence cutoffs on classification accuracy (Additional file 1: Figure S4, Figure S5, and Figure S6). As would be expected, performance improved for all tools as a function of read lengths. Taxonomer and Kraken were more sensitive to sequencing errors than BLAST and the RDP Classifier, which is not surprising given their reliance on exact k-mer matching. Nevertheless, these same analyses demonstrate that Taxonomer’s nucleotide classification algorithm is tolerant to ~5 % random error, with Taxonomer achieving greater classification accuracies than Kraken on these noisy data.

### Bacterial community composition

Since quantifying microbial community composition is a frequent goal of metagenomics studies, we compared Taxonomer’s bacterial abundance estimates to those of the RDP Classifier using recently published 16S amplicon sequencing [46, 47] and RNA-seq-based metagenomics data (Additional file 1: Table S4, Fig. 2e). The two 16S amplicon sequencing datasets were chosen as 16S-based microbiota profiling is the standard method, as data were generated with widely used sequencing instruments, and as they represent paired-end and single-end data. The RNA-seq data were chosen to demonstrate Taxonomer’s performance with shotgun metagenomics data. Taxonomer’s abundance estimates were highly correlated with RDP’s across taxonomic levels for all three datasets. Spearman correlation coefficients (ρ) were 0.96 and 0.997 (order) and 0.858 and 0.826 (genus) for 16S amplicon data as well as 0.992 (order) and 0.955 (genus) for RNA-seq (Additional file 1: Figure S7). However, Taxonomer’s average analysis times were 260- to 440-fold less than RDP’s (Fig. 2e, Additional file 1: Figure S8). Collectively, these benchmarks illustrate the importance of Taxonomer’s classification databases and the power and speed of its classification algorithm.

## Viral classification and discovery

**Fig. 3:**
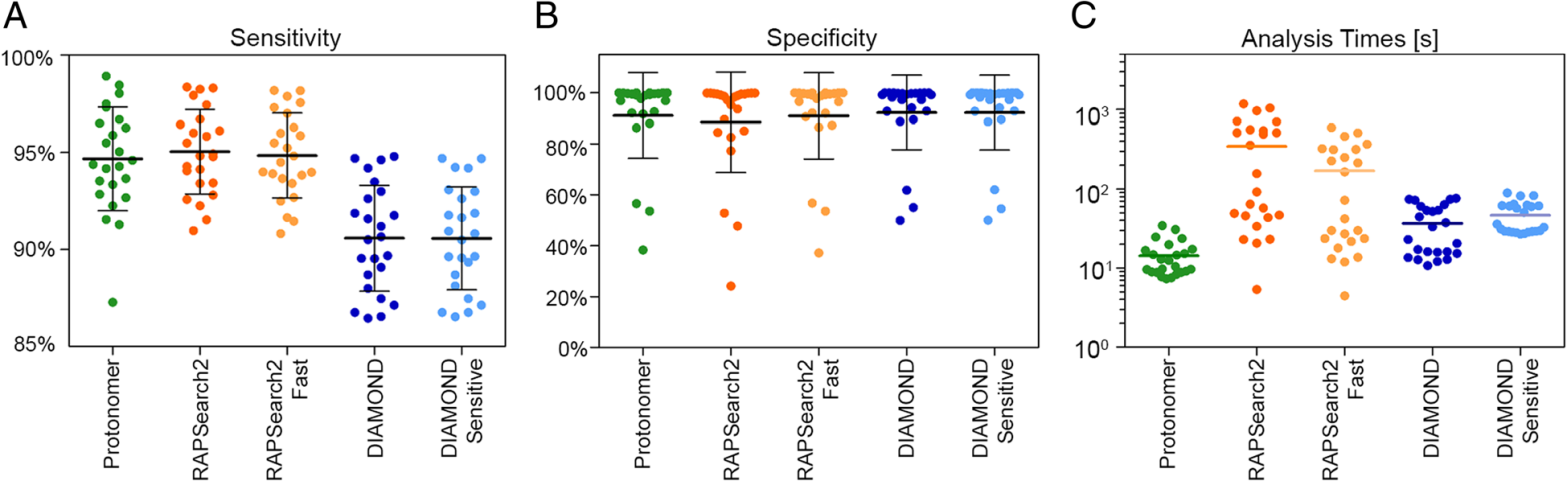
Performance characteristics of the “Protonomer” module for virus detection. RNA-Seq data from 24 samples known to harbor respiratory viruses (Additional file 1: Figure S9 and Table S11) were binned and the “viral” and “unclassified” bins were taxonomically classified by Protonomer, RAPSearch2 [36] (default and fast settings), and DIAMOND [37] (default and sensitive settings). Mean pairwise, genome-level sequence identities of the 24 respiratory viruses to reference sequences in the NCBI nt database were 93.7 % (range, 75.9–99.8 %). **a** Sensitivity. Protonomer (94.6 ± 2.7 %) and RAPSearch2 (default, 95.0 ± 2.2 %; fast, 94.8 ± 2.2 %) were more sensitive than DIAMOND (default, 90.5 ± 2.7 %; sensitive, 90.5 ± 2.7 %). **b** Specificity. Conversely, Protonomer (90.7 ± 17.1 %) and DIAMOND (default: 92.0 ± 17.1 %, sensitive: 91.9 ± 14.9 %) provided higher specificity than RAPSearch2 in default mode (88.0 ± 20.0 %). **c** Analysis times. Protonomer classifies reads faster than RAPSearch2 (24-fold compared to default mode, 11-fold compared to fast mode) and DIAMOND (2.6-fold compared to default mode, 3.3-fold compared to sensitive mode). All tools were run on 16 CPUs

Taxonomer uses reads from the “viral” and “unknown” bins (see “Methods”) for detection of viral and phage sequences (Fig. 1a, Additional file 1: Figure S1c). We compared Taxonomer’s Protonomer module to two rapid protein search tools, RAPSearch2 [36] (employed by SURPI) and DIAMOND [37] (an ultrafast, BLAST-like protein search tool), using RNA-seq data from virus-positive, pediatric NP/OP samples (n = 24). Presence of respiratory viruses was confirmed by a commercial, FDA-cleared PCR panel test or validated pathogen-specific PCR tests [40, 44]. Protonomer demonstrated the best overall performance, being more sensitive (median 94.6 %) than DIAMOND (90.5 %) and more specific (90.7 %) than RAPSearch2 (88.0 %, Fig. 3a, b). As expected, sensitivity of all tools correlated with phylogenetic distance of viral strains to reference sequences (Additional file 1: Figure S9). DIAMOND was most vulnerable to novel sequence polymorphisms. As DIAMOND does not support joint analysis of paired sequencing reads, results of the mate-pair with the lowest E-value were used, likely resulting in optimistic performance estimates. Protonomer was also the fastest of the three tools in classifying 10^4^ to 10^6^ reads/sample (median time per sample: Protonomer 14 s; DIAMOND 37 to 46 s; RAPSearch2 343 to 169 s, Fig. 3c, Additional file 1: Figure S9).

## Host mRNA expression profiling

**Fig. 4:**
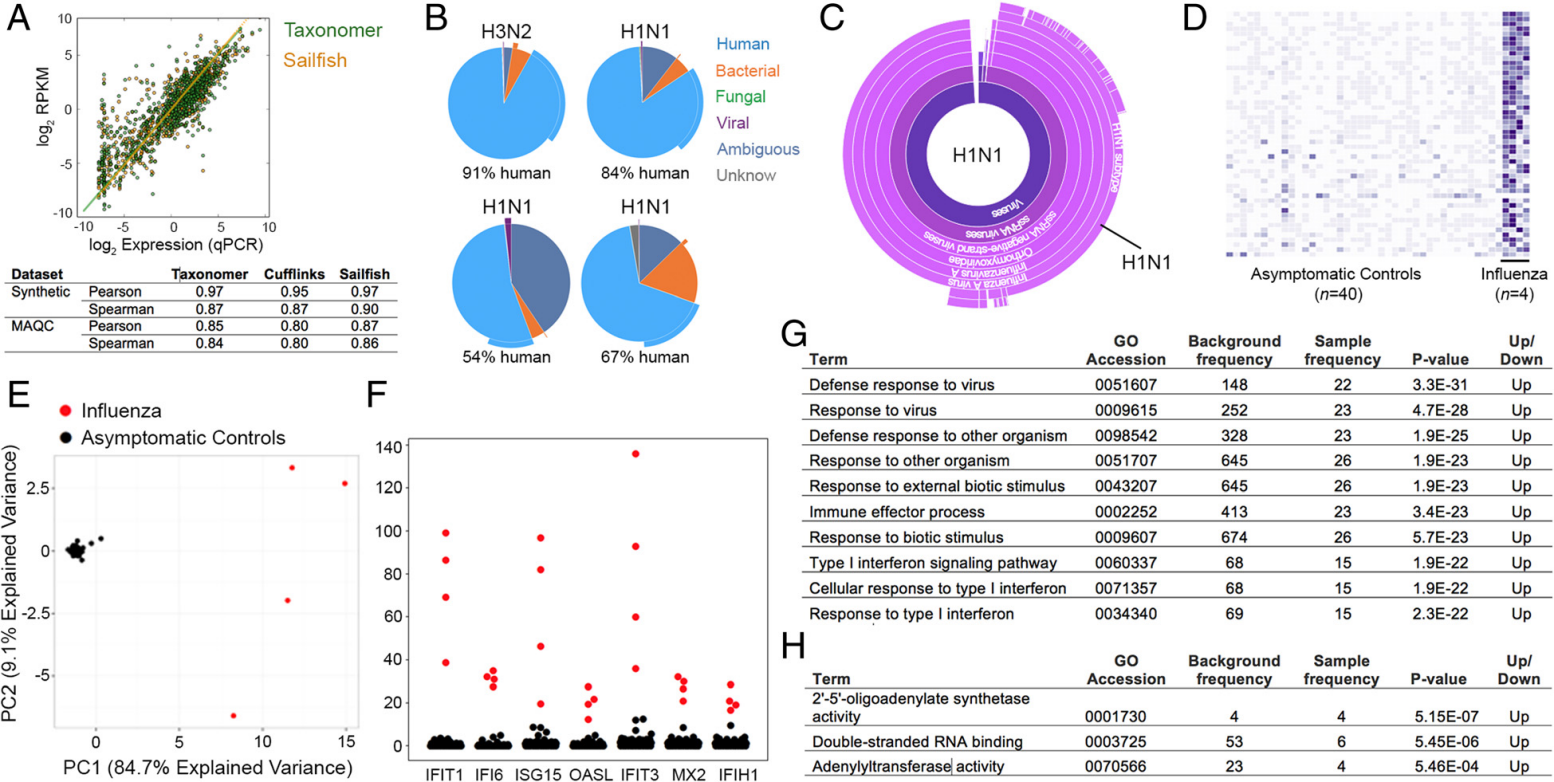
Performance characteristics of the “Classifier” module for host transcript expression profiling. **a** Published RNA-seq data from a commercially available RNA standard (MAQC, Additional file 1: Table S12) were analyzed by Taxonomer, Sailfish, and Cufflinks and estimated transcript expression was compared to data obtained by quantitative PCR (qPCR). Gene-level Pearson and Spearman correlation coefficients for RNA-seq vs. qPCR were 0.85 and 0.84 for Taxonomer, 0.87 and 0.86 for Sailfish, and 0.80 and 0.80 for Cufflinks, respectively. **b** Application of Taxonomer to metagenomic RNA-seq data from routine respiratory samples from patients with influenza infection (n = 4). **c** Classification of viral sequencing reads by Protonomer and typing of this strain as influenza A(H1N1)pdm09 (*top right sample* from **a**). **d** Differential gene-level mRNA expression profiles from four patients with influenza A virus compared to asymptomatic controls (n = 40; top 50 differentially expressed genes are shown). Expression profiles for 17 genes were significantly higher in influenza-positive patients (Additional file 1: Table S5). **e** Expression profiles for the 17 most differentially expressed genes differentiate cases from controls (principal component analysis, PC1 and PC2 explaining 93.8 % of the total variance). **f** Normalized expression levels for individual patients of seven of the top 17 genes. Gene ontology assignments for enrichment of biological processes (**g**) and molecular functions (**h**) are shown

Quantification of synthetic reads and a commercial RNA standard [48] by Taxonomer was accurate over a broad range of transcript abundance when compared to standard tools (Sailfish [38], Cufflinks [39], Fig. 4a). Indeed, Taxonomer’s accuracy was intermediate between Sailfish’s and Cufflinks’, demonstrating state-of-the-art performance. To highlight utility of simultaneous pathogen detection and transcript expression profiling, we compared [49] human mRNA expression profiles directly from respiratory samples of patients with influenza A virus infection [40, 44] (cases, n = 4) and asymptomatic controls (n = 40, Fig. 4b). PCR-confirmed Influenza A virus infections were detected in all cases (Fig. 4c). Expression profiles for 17 human genes were significantly higher in cases and clearly differentiated cases from controls (Fig. 4d–f, Additional file 1: Table S5). As expected, Gene Ontology [50] assignments for the top 50 genes demonstrated their involvement in recognizing pathogen-associated molecular patterns and in the antiviral host response (Fig. 4g and h). Most but not all of these genes are known players in the host response to viral infections (www.ncbi.nlm.nih.gov/biosystems/217173). Together, these results demonstrate the accuracy and power for discovery and diagnostic application of Taxonomer’s combined pathogen detection and host response profiling.

## Case studies

### Detection of highly pathogenic viruses

**Fig. 5:**
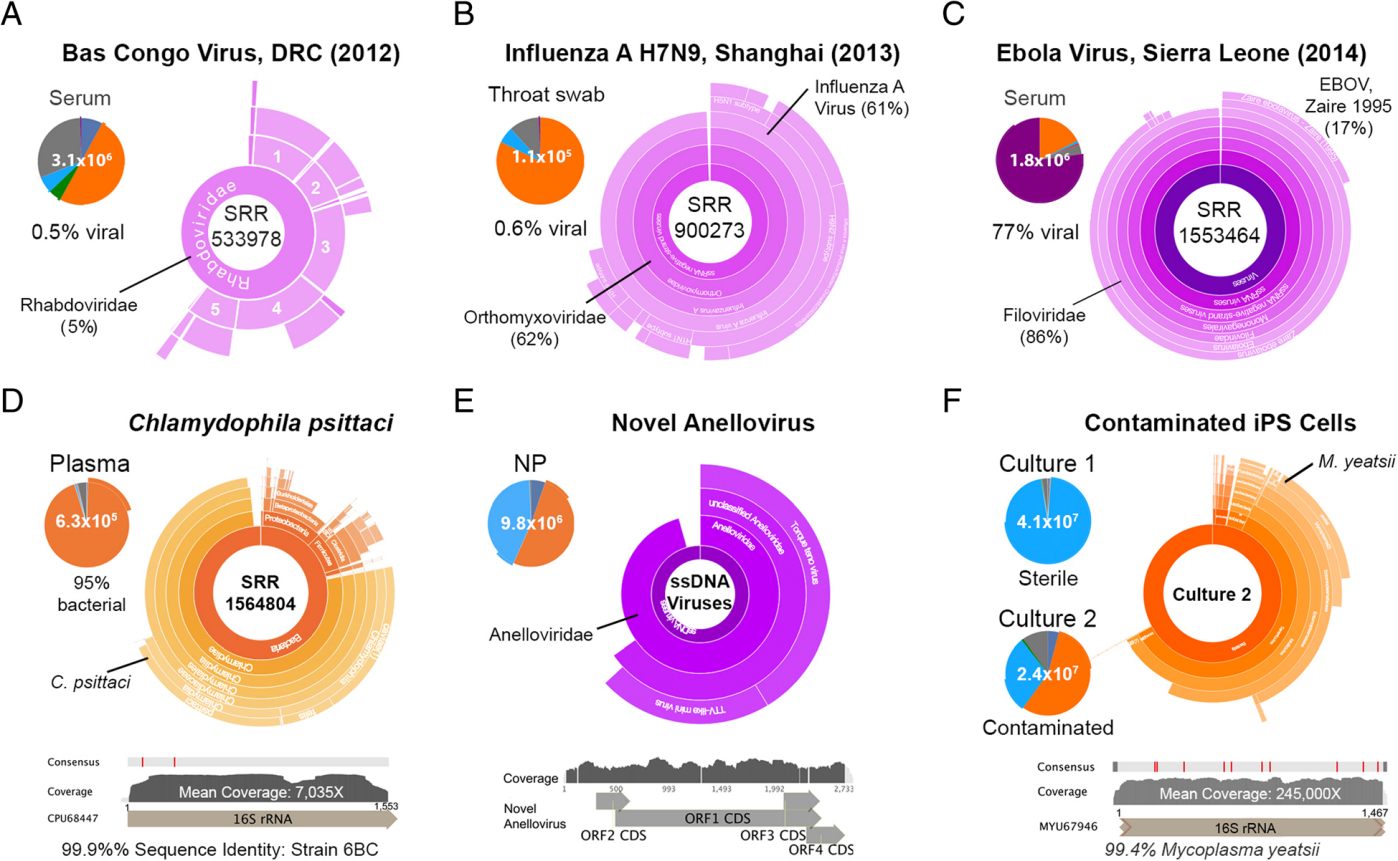
Case studies, detection of highly pathogenic viruses (**a**–**c**). To simulate viral detection and discovery in public health emergencies by Taxonomer, we removed all viral target protein sequences (as per corresponding publications [41–43]) from the reference database and analyzed published RNA-seq data with Taxonomer. The predicted viruses were detected in all cases: (**a**) novel Rhabdovirus in RNA-Seq data (SRR533978) from serum of a patient with hemorrhagic fever in the Democratic Republic of Congo (DRC), now known as Bas Congo Virus [41]; approximately 13 % of target reads from this highly divergent virus were classified at the family level (Rhabdoviridae) with genus-level assignments of Lyssavirus (1), Ephemerovirus (2), unassigned Rhabdoviridae (3), Tibrovirus (4), Sigmavirus (5); (**b**) avian influenza virus H7N9 in RNA-Seq data (SRR900273) from a throat swab of a patient in Shanghai with H7N9 infection [42]; (**c**) Ebola virus, strain Zaire 1995, in RNA-Seq data (SRR1553464) from serum of a patient with suspected Ebola virus disease in Sierra Leone [43]. Detection of previously unrecognized infections. **d** Taxonomer detected a previously unrecognized *Chlamydophila psittaci* infection (psittacosis) in plasma from a patient with suspected Ebola virus disease in Sierra Leone (SRR1564804) [43]. The 16S rRNA gene was covered a mean of 7035-fold with the consensus 16S rRNA sequence from this isolate sharing 99.9 % identity with the type strain (6BC, ATCC VR-125, CPU68447) enabling reliable identification. Positions of two single nucleotide polymorphisms are *highlighted in red*. **e** Taxonomer detected a novel Anellovirus in a nasopharyngeal swab. Forty-four reads were classified at the family level (Anelloviridae) or below. Mapping reads back to a manually constructed viral consensus genome sequence showed 14-fold mean coverage, 68.5 % pairwise nucleotide-level identity and 44–60 % predicted protein identity with TTV-like mini virus isolate LIL-y1 (EF538880.1). **f** Identification of *Mycoplasma yeatsii* contamination in RNA-seq data from cultured iPS cell (*right*) compared to non-contaminated iPS cell culture (*left*) based on read binning (*top*). High expression of rRNA is demonstrated by 32 % of RNA-Seq reads mapping to the *M. yeatsii* 16S rRNA gene (245,000X coverage, 99.4 % sequence identity with type strain GIH (MYU67946)

To demonstrate Taxonomer’s ability to detect viral pathogens in public health emergencies, we analyzed published RNA-seq data from serum of a patient with hemorrhagic fever caused by a novel rhabdovirus (Bas Congo Virus, Fig. 5a); a throat swab from a patient with avian influenza (H7N9 subtype, Fig. 5b), and plasma from a patient with Ebola virus (Fig. 5c). The presence of these viruses was confirmed in the source studies [41–43]. Even after removal of target sequences from the classification database, to simulate detection of unknown pathogens, all three viruses or close relatives were detected, thus demonstrating Taxonomer’s utility for rapid virus detection and discovery in public health emergencies.

### Detection of previously unrecognized infections 

In RNA-seq data from test-negative patients with suspected Ebola virus disease, Taxonomer detected a range of other infections confirmed by routine methods [43] (HIV, Lassa virus, Enterovirus - typed by Taxonomer as Coxsackievirus, GB virus C) (Additional file 1: Figure S10). However, Taxonomer also identified previously unrecognized bacterial infections (*Chlamydophila psittaci*, *Elizabethkingia meningoseptica*) that may have caused the patients’ symptoms (Fig. 5d, Additional file 1: Figure S11). Accuracy of these detections was confirmed manually.

Taxonomer’s power for virus discovery was demonstrated by analyzing RNA-seq data from an NP/OP sample [40] that contained a novel anellovirus with only 44–60 % predicted protein sequence identities to the most similar sequenced strain (Additional file 1: Figure S12). While 44 of 239 anellovirus reads were classified to the family Anelloviridae at the read-level (Fig. 5e), analysis of contigs assembled from all reads binned by Taxonomer as “viral” and “unknown” could be leveraged to further boost sensitivity, which resulted in detection of 4 contigs (representing all 239 reads) to the family Anelloviridae (data not shown). Presence of an anellovirus was confirmed by broad-range PCR performed at the CDC.

To demonstrate Taxonomer’s utility in quality controlling NGS data [51–56], we analyzed RNA-seq data from induced pluripotent stem cell cultures with and without *Mycoplasma* contamination (Fig. 5f). Taxonomer identified 56 % of reads as bacterial and classified the contaminant as *M. yeatsii*. The accuracy of this identification was confirmed by alignment to the 16S rRNA sequence of the *M. yeatsii* type strain GIH (MYU67946), demonstrating 99.4 % sequence identity. Lastly, Taxonomer produced highly comparable results when the same two respiratory samples positive for influenza A virus and *Mycoplasma pneumoniae* were sequenced on three popular instruments (MiSeq, HiSeq, Ion Proton, Additional file 1: Figure S13 and Table S6).

## Conclusion

In Taxonomer we have created a publically available web-service that is fast, accurate, and capable of the gamut of analyses required to take full advantage of large and complex metagenomic DNA and RNA-seq datasets that will increasingly be used to diagnose infectious diseases, profile human and environmental microbiota, investigate host mRNA expression responses, and quality control NGS datasets. Taxonomer provides these functionalities in a single web-based integrated framework without other software dependencies. This will allow the metagenomics community to explore complex metagenomics datasets without the need for bioinformatics expertise or computational resources.

It is important to note that Taxonomer’s Classifier and Protonomer modules perform taxonomic classification based on available read and reference sequence information, rather than providing a hit list of references ordered on sequence similarity as is the case for most accelerated alignment tools. The latter approach requires users to define empirical and often arbitrary classification thresholds and parse complex outputs to derive final classifications. As we have shown, Taxonomer provides more comprehensive taxonomic profiling than Kraken, and is 10–100X faster, and far more accurate than SURPI. Indeed, Taxonomer achieves accuracies on 16S amplicon data that closely approach the current standard, RDP [35]. This is made possible by Taxonomer’s comprehensive databases and its novel k-mer weighting approach, which synergize to enable reliable bacterial community profiling from RNA-seq data in which 16S sequences are highly abundant. Moreover, Taxonomer is very fast, requiring only a few minutes to carry out its broad array of analyses. On the same typical HiSeq 2500 datasets, Taxonomer is days faster than RDP, hours faster than SURPI, and within minutes of the fastest published tool, Kraken, which only provides nucleotide classification.

Taxonomer provides maximal scope for detection of known and unknown bacteria, fungi, and viruses. As the vast majority of bacteria, fungi, and viruses remain unknown [57–60], reference databases are inevitably incomplete. As we demonstrated, Taxonomer’s marker gene-based approach for bacterial and fungal identification leverages large databases that provide maximum taxonomic information, which helps avoid misclassifications pitfalls [4]. Taxonomer’s integrated means for protein-based classification further improves its sensitivity, especially for virus detection where nucleotide-based classification is of limited utility due to high mutation rates and high sequence diversity in many viral phyla. Our results demonstrate the power of Taxonomer in real-world scenarios by: (1) identifying known as well as unrecognized bacteria and viruses in previously test-negative patients; (2) by rapidly identifying microbial contamination in RNA-seq studies, which can confound transcriptional response profiles [54], lead to erroneous disease associations [51–56], or unsafe biologicals [61]; and (3) by more effective purging of host sequences prior to deposition in public databases [43]. We have also performed more detailed validation of unbiased pathogen detection by Taxonomer comparing results to a commercial multiplex PCR using respiratory samples from >100 patients [44].

Host gene expression profiling, part of Taxonomer’s integrated analysis architecture, is of growing interest for infectious diseases testing [21]. While host gene expression profiles can differentiate viral from bacterial infections using blood samples [17–19], Taxonomer enables simultaneous pathogen detection and gene expression profiling from the site of infection. This eliminates the need for a blood draw, improves diagnosis and discovery, and enables novel applications such as differentiating true infections from asymptomatic carriage, characterizing infections in immunocompromised patients, and monitoring antimicrobial treatment success.

Finally, with Taxonomer we have sought to democratize these analyses by providing a fast, interactive web-service using the publically available iobio [33] visualization toolkit. The ability to conveniently upload and rapidly analyze RNA-seq data from patient samples using personal computers and mobile devices means that results can be quickly shared and reviewed by experts, even across great geographic distances, enhancing collaborations and facilitating public health responses. As costs and turn-around times for high-throughput sequencing continue to fall and mobile sequencers become available [63], Taxonomer will enable diagnostic laboratories to analyze high-throughput sequencing data in meaningful timeframes without costly computational infrastructure or specialized bioinformatics expertise.

## Methods

### Binner module

Identifying small numbers of pathogen sequences hidden among vast numbers of host and/or microbiota-derived sequencing reads is a major algorithmic challenge for metagenomics-based pathogen detection tools. The standard approach is to use digital subtraction [64], whereby all sequencing reads are first aligned to the host’s genome sequence. This is the approach used by SURPI [32], for example. During subtraction, reads of host origin are removed. Additional subtraction steps may be used for removal of non-relevant microbial sequences, including those known to represent reagent contamination (e.g. [43, 62]) or sequencing adaptors. A greatly reduced number of presumably relevant microbial sequences are then classified by alignment to larger reference databases. Since only the remaining reads are matched with selected reference sequences, pathogens can be missed entirely if they are homologous to sequences in the subtraction database. Taxonomer overcomes this inherent limitation of digital subtraction by means of its “Binner” module (Fig. 1a), which compares each read to every reference database in parallel, assigning them to broad, non-exclusive taxonomic categories.

Taxonomer’s Binner database is created by counting unique 21 bp k-mers in different taxonomic/gene datasets using Kanalyze [65] (version 0.9.7). Each taxonomic/gene dataset represents a “bin” in which query sequences can be placed based on their k-mer content. Each database is assigned a unique bit flag that allows k-mers that belong to one or more bins to be recognized and counted. The database bins and flags are shown in Additional file 1: Table S7. The k-mer counts are merged into a binary file that contains the k-mers and the database flag. This binary file shares a similar organization to our classification databases, and is organized to optimize query speed. Reads are then assigned to the taxonomic group(s) with which most k-mers are shared. Ties are resolved as shown in Additional file 1: Table S8 and results summarized for visualization (Additional file 1: Table S9). High binning accuracy is possible because of the minimal intersections (0.47 %) of k-mer content from comprehensive human and microbial reference databases (Additional file 1: Figure S1a and b). Optimal k-mer count cutoffs were determined by Youden’s indexes and F1 scores [66] and were in the range of 3–13 (Additional file 1: Table S10, default, n = 11). To eliminate binning of reads containing adapter sequence, by default, the binner ignores k-mers present in Illumina Tru-Seq adapters. A database of External RNA Controls Consortium (ERCC) control sequences allows quantification of ERCC spike-in controls.

To demonstrate the advantage of Taxonomer’s non-greedy binning algorithm, we compared high-level taxonomic assignments made by SURPI, which employs greedy digital subtraction using sequence alignments by SNAP [67], to those of Taxonomer’s alignment-free Binner (Additional file 1: Figure S2). While high-level taxonomic assignments agree for 73.8 % of RNA-seq reads, Taxonomer assigned 16 % of reads an ambiguous origin (i.e. they match equally to multiple databases), 96 % of these were classified as human by SURPI. This was mostly due to highly conserved ribosomal and mitochondrial sequences (data not shown), but similar effects were also apparent for fungal sequences (18 % classified as human by SURPI). Taxonomer’s Binner was also able to capture more phage/viral sequences (7426) than the alignment-based method (5798), and resulted in fewer unclassified sequencing reads (3.2 % vs. 4.5 %). Consistent with lower abundance of rRNA and mtRNA sequences in DNA sequencing data, Taxonomer had many fewer ambiguous assignments (0.04 %, of which 40 % were classified as human and 59 % as viral by SURPI; overall agreement 98.7 %).

### Classifier module

Classification in Taxonomer is based on exact k-mer matching. Taxonomer uses databases that are optimized for rapid k-mer queries that store every reference in which a k-mer is found as well as an associated k-mer weight for every reference. The fundamental question for classification is how likely it is that a particular k-mer (K_i_) originates from any reference sequence, ref_i_. To answer this question, Taxonomer calculates a k-mer weight:

$$ KWre{f}_i\left({K}_i\right)\kern0.5em =\kern0.5em \frac{C_{ref}\left({K}_i\right)/{C}_{db}\left({K}_i\right)}{C_{db}\left({K}_i\right)/ Total\kern0.5em  kmer\kern0.5em  count} $$

Where C represents a function that returns the count of K_i_. C_ref_(K_i_) indicates the count of the K_i_ in a particular reference. C_db_(K_i_) indicates the count of K_i_ in the database. This weight provides a relative, database specific measure of how likely it is that a k-mer originated from a particular reference. In order to classify a query sequence, we calculate the sum of the k-mer weights for every reference that has a matching k-mer in the query sequence. Suppose that there are N possible k-mers from query sequence Q. Then, for every reference, ref_i_, that shares a k-mer with Q, the total k-mer weight for ref_i_ is:

$$ TKW\left(re{f}_i\right)\kern0.5em =\kern0.5em \underset{j\kern0.5em =\kern0.5em 1}{\overset{N}{\varSigma }}\kern0.5em  KWre{f}_i\left({K}_j\right) $$

Each read is assigned to the reference that has the maximum total k-mer weight. In the case of a tie, the query sequence is assigned to the taxonomic lowest common ancestor (LCA) [30].

### Protonomer module

We developed a mapping scheme between amino acids and their corresponding codons to facilitate mapping in protein space while using the same strategies and speed we developed for classification in nucleotide space. When the amino acid database is built for classification, Taxonomer assigns every amino acid to just one codon. This unique mapping, which we term a non-degenerate translation, is used to generate an artificial DNA sequence that corresponds to the protein sequence in the database. This DNA sequence is entered into Taxonomer’s nucleotide classification databases. Query reads are translated into all six reading frames using the same non-degenerate translation scheme used to build the database and each translated frame is then classified. K-mer weighting and read classification assignment are performed as described above. The default Protonomer database is subsets of UniRef90 and UniRef50 (see “Databases” for details). Empirically, we found a k-mer size of 30 (10 amino acids) to perform best. We chose to classify viruses in protein space because of their high mutation rates, genetic variability, and incomplete reference databases [58]. Figure 3 presents benchmark data for Protonomer and two other rapid protein search tools, RAPSearch2 [36] (employed by SURPI) and DIAMOND [37] (an ultrafast, BLAST-like protein search tool), using RNA-seq data from respiratory samples of 24 children with documented viral infections as determined by an FDA-cleared molecular test (eSensor Respiratory Virus Panel, GenMark) or targeted PCR [40] (Additional file 1: Table S11), for which complete viral genomes could be manually constructed (Geneious, version 6.1). Viral reads were defined by mapping all reads binned as “Viral” or “Unknown” to the manually constructed viral genomes. Sensitivity and specificity were determined based on detection of known viral reads (true positives) and non-viral reads (true negatives). Protonomer provides a single taxonomic identifier per read as the classification assignment, which makes interpretation of results extremely simple. Neither RAPSearch2 nor DIAMOND classify a read, instead they only provide BLAST-like alignment information. For benchmarking against RAPSearch2 and DIAMOND, the LCA of the alignment with the lowest E-value was assigned as the classification. All tools were benchmarked using the viral subset of UniRef90 as their database. Both Protonomer and RAPSearch2 process paired reads by concatenating them together with a “-” between mate pairs. DIAMOND does not support paired-end reads, so each pair was searched separately, and the hit with the lowest e-value from each read was used to make the classification assignments.

### Afterburner

To increase recovery of distantly homologous viral proteins, Taxonomer offers two options. First, unclassified reads can be further analyzed using the Afterburner module, a degenerate k-mer matching engine that employs a collapsed amino-acid alphabet (Additional file 1: Figure S14). In a manner similar to that employed by DIAMOND [37], we used k-means clustering on the BLOSUM62 matrix to generate a compressed amino acid alphabet. By using the collapsed amino acid alphabet, we are able to achieve higher sensitivity in classification with sequences that are more diverged at the expense of a higher false positive rate when compared with Protonomer (Additional file 1: Figure S14). Importantly, Taxonomer is not restricted to short reads, allowing re-analysis of resulting contigs for still greater classification sensitivity (Figs. 3 and 5).

### Host gene expression estimations

Taxonomer also uses its nucleotide classifier to assign reads to host reference transcripts. By default, these are transcripts and corresponding gene models (GTF file) from the ENSMBL human reference sequence, GRCh37.75. Empirically, we found that a k-mer size of 25 worked best for mapping reads to human transcripts. We benchmarked Taxonomer’s gene expression estimates against Sailfish’s [38] and Cufflinks’ [39] using both biological and synthetic data. To generate the benchmark data shown in Fig. 4a, we ran Taxonomer in a standalone fashion. We had Taxonomer output all ties between transcripts during the classification step; we then randomly assigned a read to a single transcript. We used these transcript level assignments to calculate gene level expression. We next employed a linear regression to correct for transcript assignment bias in a similar fashion to Sailfish. The reported correlations were then calculated using these corrected values. This level of gene expression analysis is not currently available through the web interface because of the way data are streamed; however, the results given from the web interface are a very good approximation (Spearman correlation >0.93 on a set of genes that both methods have positives counts and Spearman correlation >0.75 when the gene set is unrestricted). In the first experiment, we employed qPCR results taken from the microarray quality control study (MAQC) [48]; specifically, human brain tissue samples (Additional file 1: Table S12). We also compared performance using synthetic RNA-seq reads (2 × 76 bp, n = 15,000,000) generated with the Flux Simulator tool [68]; see Additional file 1: Table S13 for parameters. TopHat [69] was used to produce alignments for Cufflinks. Like Taxonomer, Sailfish does not need external alignment information.

### Databases

The Classifier and Protonomer databases are modular and easily constructed, consisting only of multi-fasta files with a “parent tag” on their definition lines. These tags describe each reference sequence’s immediate phylogenetic parent-taxon. Bacterial classification is based on a marker gene approach (16S rRNA gene) and the Greengenes database (reference set with operational taxonomic units, OTU, clustered at 99 %, version 13_8 [45, 70], Additional file 1: Table S7). This reference set contains 203,452 OTU clusters from 1,262,986 reference sequences. The taxonomic lineage for each OTU was used to create a hierarchical taxonomy map to represent OTU relationships. To support the OTU “species” concept, the taxonomy was completed for ranks in the taxonomic lineage that had no value. Unique dummy species names from the highest taxonomic rank available were used to fill empty values. Versions of the Greengenes database were formatted for use within BLAST, the RDP Classifier, and Kraken. Fungal classification is also based on a marker gene approach (internal transcribed spacer, ITS, rRNA sequences) and the UNITE database [60] (version sh_taxonomy_qiime_ver6_dynamic_s_09.02.2014, Additional file 1: Table S7). This reference set contains 45,674 taxa (species hypothesis, SH) generated from 376,803 reference sequences with a default-clustering threshold of 98.5 % and expert taxonomic curation. Dummy names were created for ranks that had no value. Versions of the unite database were formatted for use with BLAST, the RDP Classifier, and Kraken. Viral classification and discovery. The virus classification database consists of the viral subset of UniRef90 [71] (release 2014_06) combined with the bacterial subset of UniRef50 (release 2015_03). The viral protein database was reduced to 289,486 viral sequences based on NCBI taxonomy. Phage sequences were separated, leaving a total of 200,880 references for other viruses. NCBI taxonomy was used to determine the sequence relationship. For viral classification and discovery benchmarks shown in Fig 3a–c and for contig-level classification, only the viral subset of UniRef90 was used.

### Additional classification databases

For testing purposes, additional bacterial classification databases were constructed from RefSeq (identical to Kraken’s full database; n = 210,627 total references; n = 5242 bacterial references, using NCBI taxonomy) and the complete ribosomal database project databases download on 24 September 2014 (n = 2,929,433 references, using RDP taxonomy).

### Database construction

Databases are constructed to maximize query speed. K-mers are stored in lexicographical order and k-mer minimizers are used to point to blocks of k-mers in the database. Once a block of k-mers is isolated, a binary search is used to complete the query. This scheme provides extraordinary query speeds, as demonstrated by Wood and Salzberg [30]. We employ the same basic database layout as Kraken, with the important difference that instead of storing just the LCA of a k-mer, we also store the k-mer count and every reference (up to an adjustable cutoff) with associated k-mer weight. Detailed information about the database format and layout is available upon request.

### Gene classification protocols

We extracted reference sequences from widely used, curated public databases for benchmark experiments [12]. These reference sequences were used to generate synthetic read datasets having a variety of read-lengths and error rates using wgsim (https://github.com/lh3/wgsim). PCR-amplified 16S rRNA gene sequences from two metagenomics studies on stool [47] and the home environment [46] were also used. The analysis was limited to taxa with relative abundance >0.1 % per sample (10 random samples were selected from each study).

#### Bacterial 16S rRNA

From the SILVA 119 non-redundant small-subunit ribosomal sequence reference database [12], we extracted bacterial reference sequences between 1200 and 1650 bp of length and excluded references annotated as cyanobacteria, mitochondria, and chloroplasts. Only high quality references without ambiguous bases, alignment quality values >50 %, and sequence quality >70 % were included. All the above values are reported by SILVA. Percent identity to the closest Greengenes OTU was determined by MegaBLAST [72] using hits with a query coverage >80 %. Synthetic reads (100 bp single-end, 100 bp paired-end, 250 paired-end) were generated from these reference sequences at 5× coverage.

#### Fungal ITS

To test the accuracy of identifying fungal ITS sequences that are not represented in the UNITE database [60], we utilized the UNITE_public_dataset (version_15.01.14). Percent identity to the closest UNITE species hypothesis (SH, OTUs clustered at 98.5 %) was determined by MegaBLAST using hits with a query coverage >80 %. Synthetic reads (250 bp single-end) were generated from these reference sequences at 5× coverage. Due to the variable length of ITS sequences (mean 585 bp, range 51–2995 bp, n = 376,803), paired-end sequences were not generated.

### Classification criteria for reference methods

#### BLAST

Default MegaBLAST parameters were used. Top scoring references were identified and used to assign OTUs/SHs. Multiple OTUs/SHs were assigned to synthetic reads when more than one OTU/SH reference shared 100 % identity. If no OTU/SH had 100 % identity to a read, then all OTUs within 0.5 % of the top hit were assigned to the read. The taxonomy of the assigned OTUs/SHs was compared and the highest rank in common was used to assign a taxonomic value to the read. The percent identity was used to determine the assignment of the highest taxonomic rank. Sequence reads with >97 % identity to a reference were assigned to species, >90 % identity to genus, and <90 % to family when lineage information was available at this rank.

#### RDP classifier

RDP classifier analyses were performed on a local server (see below). Classifications were resolved to the rank with a minimum confidence level of ≥0.5.

#### Kraken

Kraken analyses were performed on a local server (see below). Kraken reports the taxon identifier for each read’s final taxonomic assignment. An accessory script (Kraken-filter) can be used to apply confidence scores, although we found this value had little impact on results of our benchmarks. The effect of applying different confidence scores is shown in Additional file 1: Figure S6.

#### SURPI

SURPI analyses were performed using an Amazon EC2 instance through the published Amazon Machine Image. SURPI reports the best hit for its mapping tools (SNAP [67], RAPSearch2), which were used for comparison.

### Taxonomer implementation

Taxonomer was written in C with Python bindings through Cython. An implementation of Taxonomer that contains the entire pipeline functionality was written in C and drives the iobio web interface.

### Server specifications

Benchmarking was performed on a machine with Red Hat Linux, 1 TB of RAM, and 80 CPUs. Number of CPUs was restricted to 16 unless otherwise noted.

### Web-service and visualization

Taxonomer is publically available as a web-service built upon the iobio framework [33]. It is available at taxonomer.iobio.io. Complex metagenomic data can be processed quickly and effectively interpreted through web-based visualizations. Figure 1b illustrates the interface. As reads are being streamed to the analysis server, a pie chart is presented summarizing the results of the binning procedure. When one of the bacterial, fungal, viral, or phage bins of the pie chart is selected, the results of the Classifier/Protonomer modules are displayed in a sunburst visualization. Additional information is provided at the top of the web page about how many reads were sampled, the number of reads classified, and the detection threshold. The detection threshold informs a user about how abundant a particular organism must be in order to be detected with the number of reads sampled. This provides an indicator of the sensitivity of detection in the sample. In addition, a slider allows the user to select an absolute cutoff for the minimum number of reads required in order to be displayed in the sunburst.

### DNA and RNA-seq of patient samples

#### Nucleic acid extraction

Samples (75–200 μL) were extracted using the QIAamp Viral RNA extraction kit (Qiagen). Extraction was carried out as described by the manufacturer with the exception of the AW1 washing step. For this step, 250 μL of AW1 wash buffer was added to the QIAamp Mini column before centrifugation at 8000 rpm. Then, 80 μL of DNase I mix (Qiagen) containing 10 μL of RNase-free DNase I and 70 μL of Buffer RDD was added to the column for on column DNase digestion. After incubation at room temperature for 15 min, an additional 250 μL of AW1 was added to the column before centrifugation at 8000 rpm. The manufacturer’s suggested protocol was continued at this point with column washing using Buffer AW2. After all washing steps, RNA was eluted in 60 μL of water. Extraction for total DNA was performed using 75–200 μL of sample with the DNeasy Blood and Tissue Kit (Qiagen) according to the manufacturer’s instructions. DNA was eluted in 200 μL of nuclease-free water.

#### Depletion of human DNA

Microbial DNA was enriched with NEBNext Microbiome DNA Enrichment Kit (NEB). Briefly, MBD2-Fc-bound magnetic beads were prepared by combining 3 μL of MBD2-Fc protein with 30 μL of Protein A Magnetic Beads per sample and placing the mixture in a rotating mixer for 10 min at room temperature before washing with 1× Binding Buffer. Extracted DNA (200 ng in 200 μL) was added to 50 μL 5× Binding Buffer. The resulting 250 uL were added to MBD2-Fc-bound magnetic beads for 15 min at room temperature with rotation. The enriched microbial DNA was cleaned-up with Agencourt AMPure XP Beads (Beckman Coulter).

#### Library generation

For HiSeq and MiSeq sequencing, indexed cDNA libraries were produced from extracted RNA using the TruSeq RNA Sample Prep Kit v2 (Illumina) omitting poly-A selection. RNA was dried and resuspended in 19.5 μL of Elute, Prime, Fragment Mix. The remainder of the library preparation was conducted per manufacturer’s instructions. Before library generation from DNA, enriched microbial DNA was fragmented with the Covaris S2 Ultrasonicator using intensity 5, duty cycle 10 %, and 200 cycles/burst for 80 s all at 7 °C. Libraries generated from fragmented enriched microbial DNA were prepared using the KAPA Hyper Prep Kit (KAPA Biosystems) according to the manufacturer’s instructions. PCR cycles used for library amplification were dependent upon the amount of input DNA and 13 cycles were used for these experiments. Libraries were quantitated by qPCR using the KAPA SYBR FAST ABI Prism qPCR Kit (KAPA BioSciences) and the Applied Biosystems 7900HT Fast Real-Time PCR System (Applied Biosciences). Library size was determined with the Agilent High Sensitivity DNA Kit and Agilent 2100 Bioanalyzer. After pooling of indexed sequencing libraries, a second qPCR and bioanalyzer run was performed to estimate the final concentration before sequencing. For Ion Proton sequencing, indexed cDNA libraries were produced from extracted RNA using the SMARTer Universal Low Input RNA Kit (Clontech) with numbers of PCR cycles in the range of 10–15 based on RNA yield.

#### Sequencing

Pooled sequencing libraries were analyzed on a HiSeq 2500 (2 × 100 bp), MiSeq (2 × 250 bp, both Illumina), or Ion Proton (median read length 139 bp, Life Technologies) instruments according to manufacturers’ protocols.

### Statistical analyses

For gene expression analyses, we report both the Pearson and Spearman correlations as was done before [38]. Correlation coefficients were calculated using the scipy library for python. The Pearson correlation of the log transformed gene expression estimates necessitates the removal of any genes whose estimated expression is 0. The log transform prevents outliers from dominating the correlation. We also report the Spearman correlation, for which the log transform is not as necessary since it is a correlation based on ranks. Thus the exclusion of genes with estimates of 0 can be avoided.

## Additional file

Additional file 1: Supplementary results. (DOCX 12212 kb)
