# Supplementary material for: Taxonomer: an interactive metagenomics analysis portal for universal pathogen detection and host mRNA expression profiling
Source: Genome Biol. 2016 May 26;17:111. doi: 10.1186/s13059-016-0969-1 (PMC4880956; doi:10.1186/s13059-016-0969-1)
Supplement: Additional file 1: — Supplementary results. (DOCX 12212 kb) [file 13059_2016_969_MOESM1_ESM.docx]

**
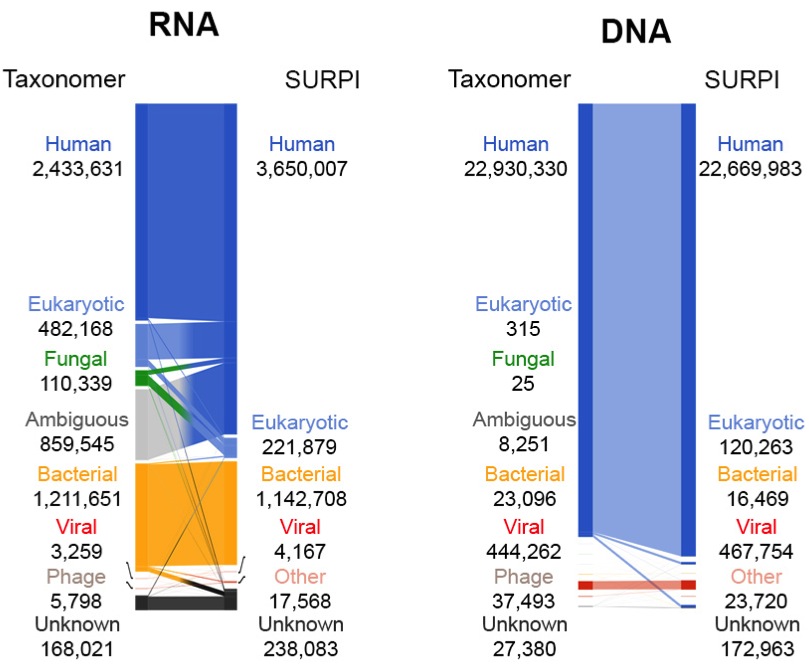
**

**Supplementary Figure 1**. The ‘Binner’ module calculates the number of k-mers shared between a sequencing read and each of the binning databases prior to read assignment (parallel approach). A sequential strategy introduces bias since reads may be assigned based on an earlier, but less ideal match. This bipart graph shows agreement between read binning by the ‘Binner’ module versus SURPI’s assignments, which are based upon sequential subtraction. These data come from one of the 33 pediatric respiratory tract samples shown in supplemental Fig 1D (RNA) and an additional nasopharyngeal sample (DNA). Of reads classified as human by SURPI, 1% was classified by Taxonomer as fungal, to lower resolution (11%), or cannot confidently be differentiated between closely related bins (23%) when using a simultaneous binning strategy. Consistent with lower abundance of rRNA and mtRNA sequences in DNA sequencing data, Taxonomer had many fewer ambiguous assignments (0.04%, of which 40% were classified as human and 59% as viral by SURPI; overall agreement 98.7%).

**
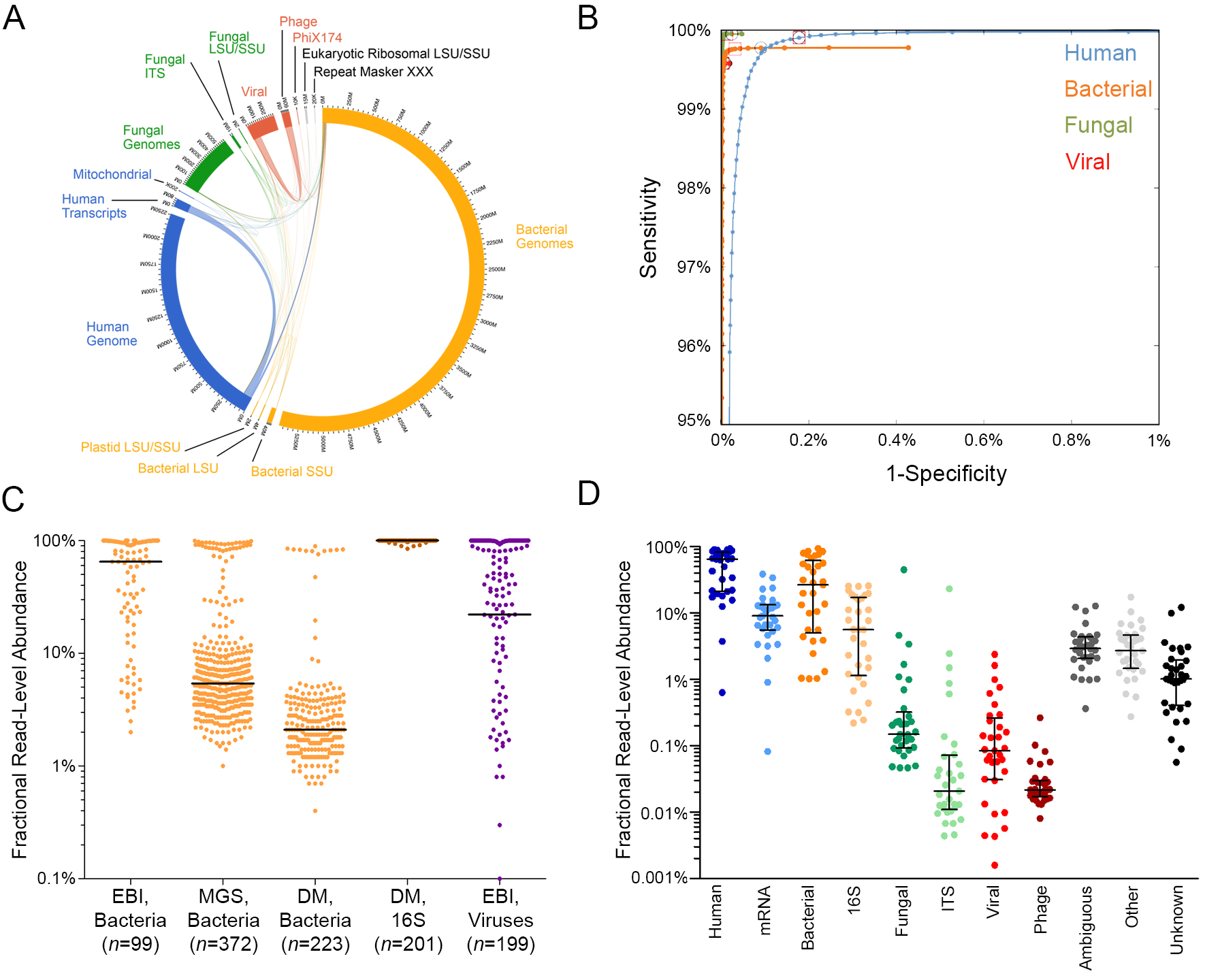
**

**Supplementary Figure 2. Performance characteristics of the ‘Binner’ module. (a)** Intersections of default k-mer databases used by the ‘Binner’ module are minimal, which provides the basis for highly specific read binning. The total proportion of intersecting k-mers between databases that are not complete subsets of larger databases (e.g. ‘phage’ database is a subset of the ‘virus’ database) is 0.47%. The number of total k-mers in each of the 15 k-mer databases used for read binning is provided in millions. The widths of the cords that connect different sections indicate the number of intersecting k-mers. (**b**) Receiver operator characteristics curves for classification of human and microbial sequences by the ‘Binner’ module. A total of 1x10^6^ synthetic 100bp reads (80% human, 10% bacterial, 5% fungal, 1%viral, and 4% from parasites; 1% error rate) were analyzed with the ‘Binner’ module and interpreted for correct bin assignment to calculate sensitivity and specificity using minimum k-mer count thresholds for read binning ranging from 1 to 40. Boxed and circled thresholds represent optimal cutoffs as determined by F1 score and Youden’s index^1^, respectively (see **Supplementary Table 2**). **(c)** Sensitivity for binning of bacterial and viral reads can be low for phylogenetically distant species. Synthetic bacterial and viral reads were generated from single-cell sequencing-based draft bacterial genomes^2^, bacterial genome scaffolds derived from metagenomic sequencing data^3^, and recently published genome sequences. Sensitivity for correct binning (vs. assignment as ‘Unknown’) can be low for bacteria (median 2.1%, 5.4%, and 64.9%, respectively) and viruses (median 22.1%, 0% for *n*=56 of 199 viral genomes) not represented in the Binner database. In contrast, 16S sequences from the same unrepresented bacteria are almost always correctly binned (median 100%). This highlights the conservation of the 16S rRNA marker gene and the greater completeness of reference databases. As a result, organisms are still identified as present within the sample and can be placed within phylogenetic context. **(d)** Relative read abundance of different taxonomic bin as determined by the ‘Binner’ module for 33 pediatric respiratory tract samples positive for at least one respiratory virus^4^, including median and interquartile range (IQR) for each bin (6.3x10^6^±2x10^6^ reads/sample). Relative abundances vary greatly for all bins but reached almost 4 orders of magnitude for the viral and fungal bins. A median of only 1% (IQR 0.4-2%) of reads could not be assigned a bin (unknown). A median of 9% of reads were derived from human mRNA, supporting the idea that host transcript expression profiling can be performed using total RNA-seq from nasopharyngeal samples.

**Supplementary Figure 3. Query sequences not represented in the reference database cause false-positive and false-negative classifications. (a)** Read-level classification accuracy for synthetic reads simulated (20X coverage) from SILVA^8^ references (*n*=10,000) with identical representation in the reference database as classified by BLAST, the RDP Classifier, Kraken, and Taxonomer. Note, while only 84.2% (BLAST), 85.2% (RDP), 64.9% (Kraken), and 83.7% (Taxonomer) of reads are classified to the species level (an effect of highly conserved regions of the 16S gene not allowing species-level assignment), false-positive rates are minimal for all classification algorithms, 0.4% (BLAST), 0.7% (RDP), 0.02% (Kraken), and 0.1% (Taxonomer). **(b)** Panel b shows the same analysis with SILVA references (*n*=10,000) for whom highly similar, but non-identical references (97% to 98.99% pairwise sequence identity based on full-length MegaBLAST) are present in the reference database. Proportions of reads with species-level classification drop to 39.1% (BLAST), 49.0% (RDP), 26.9% (Kraken), and 47.4% (Taxonomer) and 5.3% (BLAST), 5.1% (RDP), 10.2% (Kraken), and 13.7% (Taxonomer) of reads are classified to taxa that are different from the source of the synthetic reads. **(c)** This effect is even more pronounced for synthetic reads simulated from SILVA references (*n*=10,000) that only share 90% to 96.99% pairwise sequence identity with the closest match in the reference database (based on full-length MegaBLAST). Species-level classification becomes impossible by commonly used definition but even genus-level classification drops to 33.0% (BLAST), 40.8% (RDP), 32.1% (Kraken), and 38.8% (Taxonomer). At the species-level, 22.1% (BLAST), 51.5% (RDP), 55.7% (Kraken), and 66.4% (Taxonomer) of reads are assigned to taxa other than those they were simulated from. All studies were performed with 250bp paired-end 16S rDNA reads simulated at 20X coverage from randomly selected SILVA references with no error.

**A B**

**Supplementary Figure 4**. Read-level (top) and taxon-level (bottom) bacterial classification accuracy of BLAST, the RDP Classifier, Kraken, and Taxonomer (all tools with the Greengenes 99% OTU database) using (**A**) 100bp single-end and (**B**) 100bp paired-end 16S rDNA reads simulated at 5X coverage from 1,013 randomly selected SILVA references with ≥97% sequence identity to reference sequences (see methods). Performance of Taxonomer is comparable to the RDP Classifier and superior to Kraken; given the applied criteria, BLAST is less sensitive but more specific.

**Supplementary Figure 5. Impact of sequencing error rates**. Family, genus and species level classification accuracy for BLAST, the RDP Classifier, Kraken and Taxonomer using the same read-length and database across error rates of 0.01%, 0.1%, 1%, 5%, and 10%.

**Supplementary Figure 6**. Effect of Kraken’s classification using confidence cutoffs on read-level classification accuracy. Applying Kraken’s confidence cutoffs [0.0, 0.02, 0.04, 0.06, 0.08, 0.1, 0.2, 0.3, 0.4, 0.5] did not improve overall performance.

**Supplementary Figure 7.** Taxonomer classifies bacterial 16S rRNA reads at >200-fold increased speed compared to the RDP Classifier^9^ while providing highly comparable bacterial community profiles. **(a)** Bacterial community profiling using RNA-Seq-based shotgun metagenomics with pediatric nasopharyngeal and oropharyngeal swab samples (*n*=20) with Taxonomer and the RDP Classifier at the genus-level. Taxonomer provides highly comparable community profiles at >200-fold increased speed (Spearman correlation coefficient: ρ=0.955 for 2x100bp reads); average of 1,630,923 2x100 bp reads/sample; average run times were 27.4 minutes (Taxonomer) versus 120.7 hours (RDP Classifier) on 1 CPU. **(b)** RNA-Seq metagenomics results (as in panel a) were also analyzed by Kraken^10^ using the Greengenes 99% OTU reference database. Correlation with abundance estimates based on the RDP Classifier were weaker (Spearman correlation coefficient: ρ=0.891 2x100bp reads); average run times were 42 seconds/sample. **(c)** 16S rRNA gene amplicon sequences of variable region 4 from 2 published data sets generated on HiSeq2000^11^ (dark green, 1x150bp reads) and MiSeq^12^ instruments (light green, 2x150 reads). Correlation of abundance estimates (limited to taxa with relative abundance >0.1% per sample) are shown for Taxonomer and the RDP Classifier (Spearman correlation coefficients: ρ=0.858 for 1x150bp reads^12^ and ρ=0.826 for 2x150bp reads^11^). The average number of reads per sample was 44,685 and the average processing times (using 1 CPU) were 1:28 minutes for Taxonomer and 7.9 hours for the RDP Classifier. **(d)** 16S rRNA gene amplicon sequences (as in panel c) were also analyzed by Kraken using the Greengenes 99% OTU reference database. Correlation with abundance estimates based on the RDP Classifier were weaker (Spearman correlation coefficient: ρ=0.697 for 1x150bp reads^12^ and ρ=0.810 for 2x150bp reads^11^); average run times were 2.5 seconds/sample.

**Supplementary Figure 8.** Analysis times for the RDP Classifier (R), Taxonomer (T), and Kraken (K) for classification of samples shown in Supplementary Fig. 9.


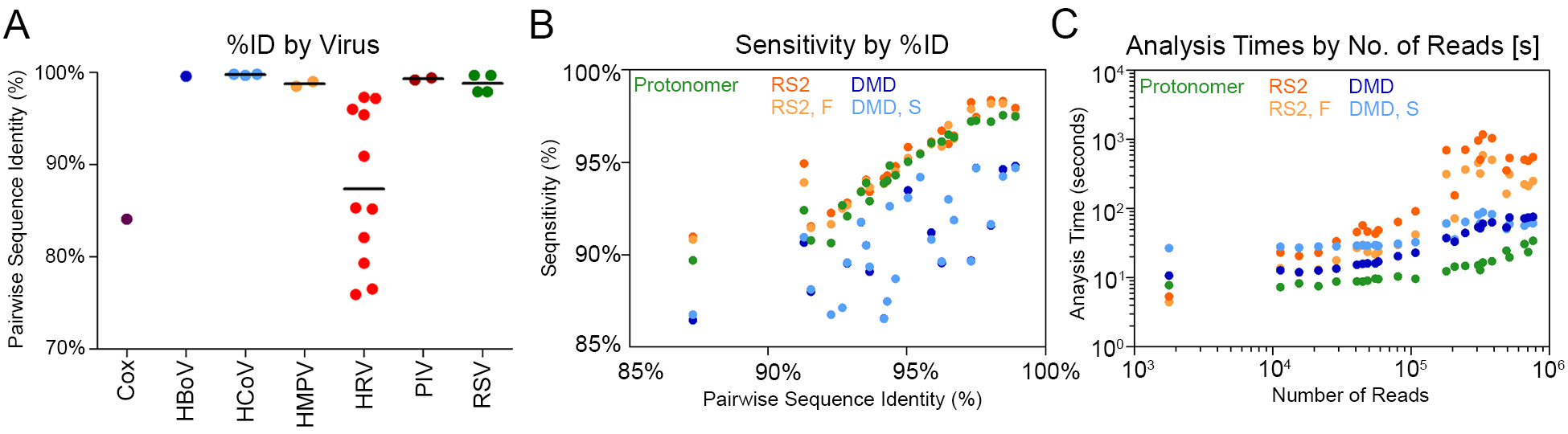


**Supplementary Figure 9. (a)** Nucleotide sequence identity of viral consensus sequences from 23 respiratory samples used for Protonomer benchmarking to the best match in the NCBI nt database (see Fig 4, see Supplementary Table 5 for details; Human coronavirus, *n*=3; Coxsackievirus, *n*=1; human bocavirus, *n*=1; human metapneumovirus, *n*=2; rhinovirus, *n*=10; parainfluenza virus, *n*=2; and respiratory syncytial virus, *n*=4). Virus-positive samples were selected to represent a range of pairwise identities. **(b)** Sequencing reads were binned and the ‘viral’ and ‘unclassified’ bins were taxonomically classified by Protonomer, RAPSearch2^5^ (default and fast settings), and DIAMOND^6^ (default and sensitive settings, see **Figure 3**). The greater sensitivity for correct read-level identification of viral reads of Protonomer (mean 94.6±2.7%) and RAPSearch2 (default, 95.0±2.2%; fast, 94.8±2.2%) compared to DIAMOND (default, 90.5±2.7%; sensitive, 90.5±2.7%) is apparent over the entire range of pairwise identities. True viral reads were determined by mapping^7^ of all reads to a manually constructed viral consensus genome sequence for each sample. **(c)** Analysis times for Protonomer, RAPSearch2, and DIAMOND were tested on the same samples, which required analysis of between ~2,000 and ~1,000,000 ‘viral’ + ‘unknown’ sequences. Protonomer classifies reads faster than RAPSearch2 and DIAMOND across this range. All tools were run on 16 CPU.
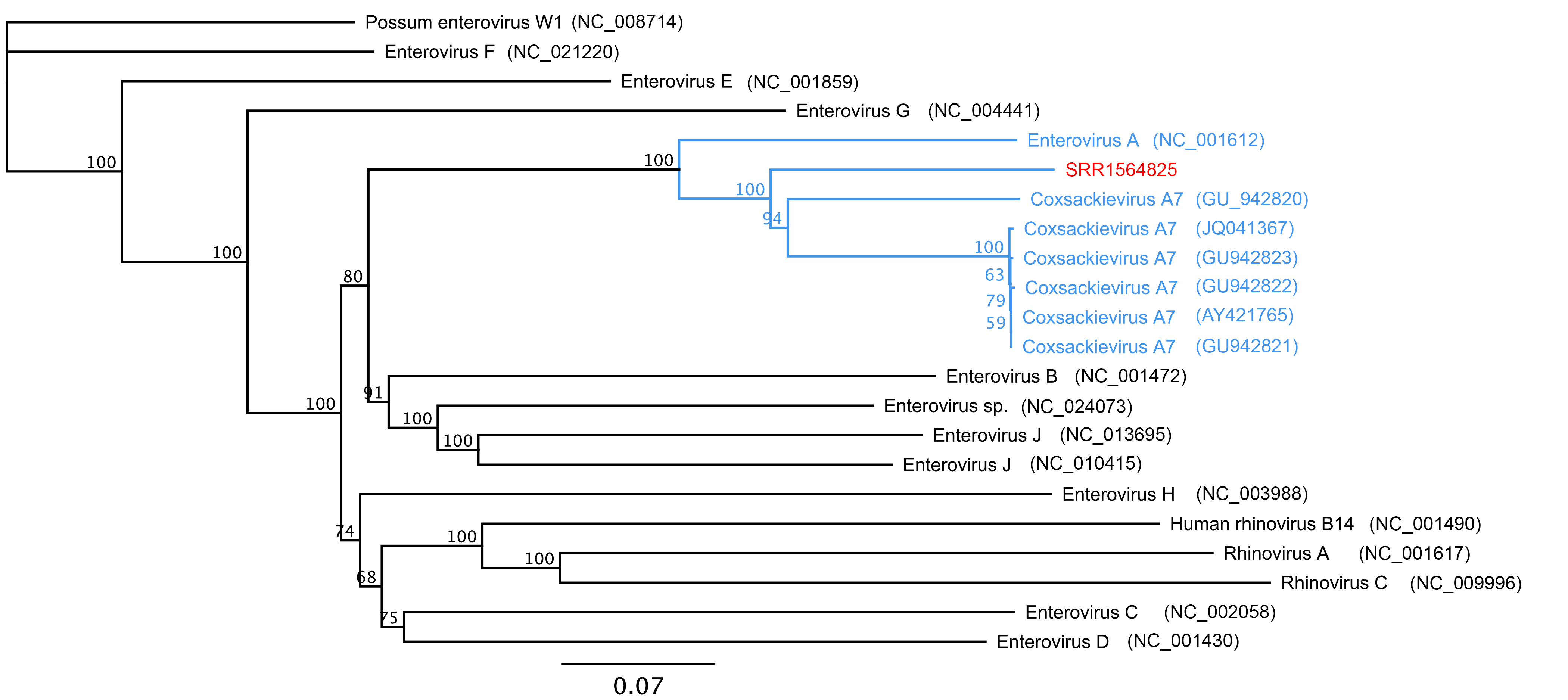


**Supplementary Figure 1**0. Phylogenetic tree of based on the consensus sequence of the Coxsackievirus strain detected in SRR 1564825 (highlighted in red) and related reference sequences (RefSeq strains for all Enterovirus species (black font) and all full length sequences for Coxsackievirus A7, blue font). The multiple sequence alignment was performed with MUSCLE and a Neighbor-Joining consensus tree was constructed using the method of Tamura-Nei with 1,000 replicates.


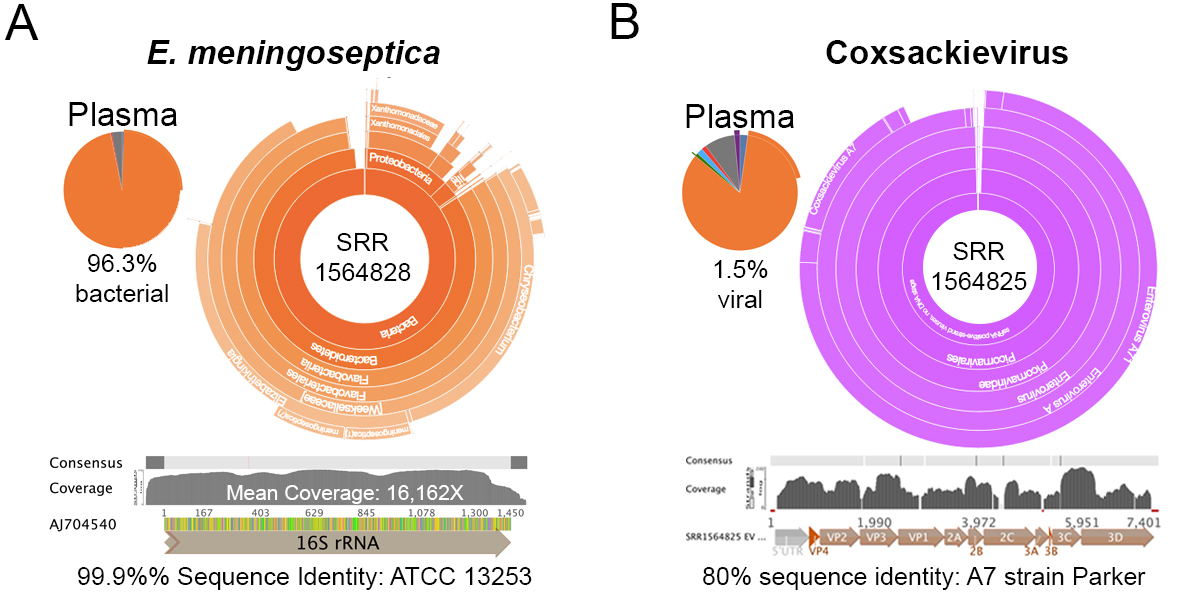


**Supplementary Figure 11. (a)** Taxonomer detected *Elizabethkingia meningoseptica* in sample SAMN03015718 (SRR1564828)^13^. Mean coverage of the 16S rRNA gene was 16,162-fold and the consensus sequence shared 99.9% nucleotide sequence identity with the type strain of *E. meningoseptica* (AJ704540, ATCC 13253). *E. meningoseptica* is a ubiquitous gram-negative bacterium that characteristically causes meningitis or sepsis in newborns but also immunocompromized adults. **(b)** Taxonomer classified a reported Enterovirus as Enterovirus A in plasma from a patient with suspected Ebola virus disease in Sierra Leone (SRR1564825)^13^. Mean sequencing depth was 162X covering 96% of the reference sequence (AY421765). Analysis of a manually constructed viral consensus genome sequences identified the strain as sharing 80% nucleotide sequence identity with Coxsackie virus A7, strain Parker.

**
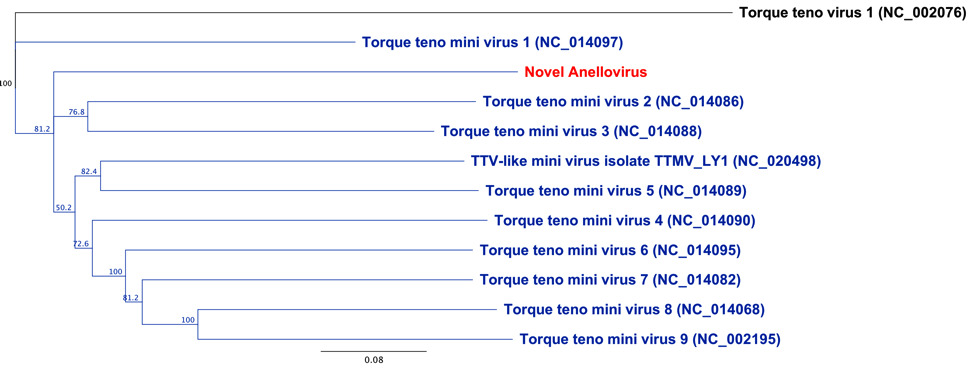
**

**Supplementary Figure 12.** Phylogenetic tree of consensus sequence of novel Anellovirus (**Fig. 5b**) with reference sequences for Torque teno mini viruses. Torque teno virus 1 is shown as outgroup.


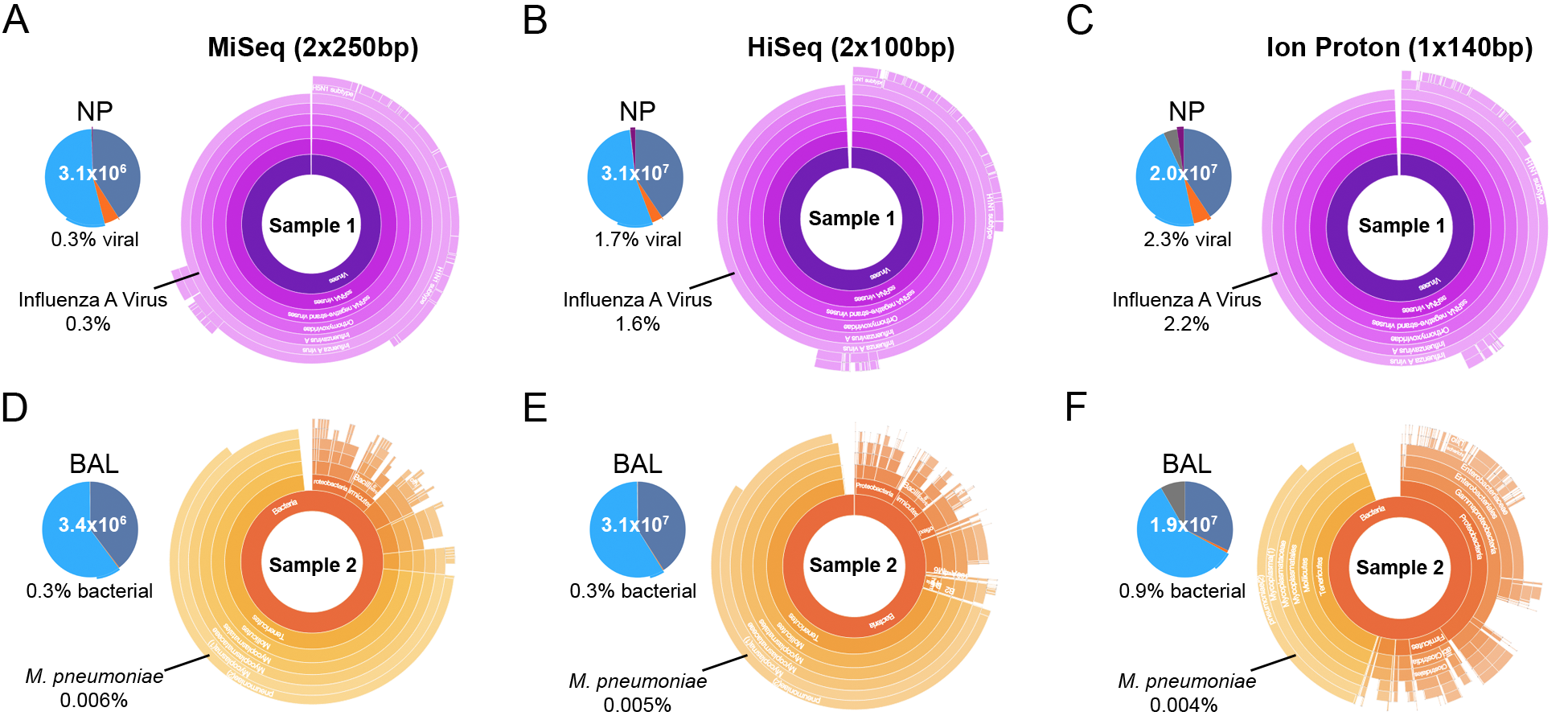


**Supplementary Figure 13. (d)** Taxonomer is compatible with different sequencing protocols, recovering similar proportions of viral (influenza A, 0.43% to 0.55% of all reads, a-c) and bacterial (*Mycoplasma pneumoniae*, 16S rRNA sequences representing 0.004% to 0.006% of all reads, d-f) pathogen sequences when sequencing samples on 3 commonly-used sequencers (illumina MiSeq and Hiseq, Thermo Scientific Proton) with 2 different library preparation methods. Samples were known to be positive for influenza A(H1N1)pdm09 and *M. pneumoniae* based on PCR test validated in our national reference laboratory for diagnostic application.

**
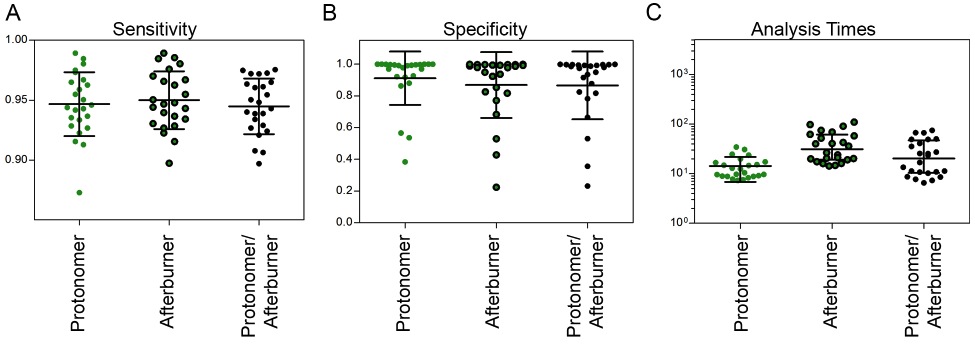
**

**Supplementary Figure 14 (a)** RNA-Seq data from 23 samples known to harbor respiratory viruses (human coronavirus, *n*=3; Coxsackievirus, *n*=1; human bocavirus, *n*=1; human metapneumovirus, *n*=2; rhinovirus, *n*=10; parainfluenza virus, *n*=2; and respiratory syncytial virus, *n*=4) were binned and the ‘viral’ and ‘unclassified’ bins were taxonomically classified by Protonomer, Afterburner, and Protonomer followed by Afterburner analysis of previously unclassified reads (samples as in Fig 4, see Supplementary Figure 9 and Supplementary Table 5). Protonomer (94.6±2.7%) and Afterburner (94.5±2.3%) had comparable sensitivity while their combination was slightly more sensitive (95.0±2.4%). **(b)** Conversely, Protonomer (91.1±16.8%) was slightly more specific than Afterburner (86.6±21.4%) and a combination of the two tools (86.8±20.7%). True viral reads were determined by mapping^7^ of all reads to a manually constructed viral consensus genome sequence for each sample. **(c)** Mean analysis times were 14.3±7.5 seconds (Protonomer), 27.4±21.5 seconds (Protonomer/Afterburner), and 41.7±28.7 seconds (Afterburner). All tools were run on 16 CPU.

**Supplementary Table 1. Effect of joint analysis of mate pairs on read binning.** Sample 2 from **Table 1** was analyzed with the ‘Binner’ module either using only read 1, only read 2, or analyzing both reads jointly (after concatenation, see methods). Concatenation of mate pairs results in fewer reads with unknown (-13%) and ambiguous bin assignment (-19%) compared to results based on read 1 alone. The largest relative change is seen for phages (+58%), bacteria (+19%), fungi (+18%), and ‘Other’ (+17%).

|  | **Read 1** | |  | **Read 2** | |  | **Concatenated** | | **Change** |
| --- | --- | --- | --- | --- | --- | --- | --- | --- | --- |
| **Bin** | **Reads** | **%** |  | **Reads** | **%** |  | **Reads** | **%** |  |
| **Human** | 1,907,759 | 32.2 |  | 1,902,495 | 32.1 |  | 1,944,049 | 32.8 | +2% |
| **Bacterial** | 952,402 | 16.1 |  | 953,957 | 16.1 |  | 1,134,751 | 19.1 | +19% |
| **Fungal** | 274,232 | 4.6 |  | 274,800 | 4.6 |  | 324,874 | 5.5 | +18% |
| **Viral** | 4,792 | 0.1 |  | 4,799 | 0.1 |  | 5,470 | 0.1 | +14% |
| **Phage** | 1,292 | 0.0 |  | 1,434 | 0.0 |  | 2,041 | 0.0 | +58% |
| **Ambiguous** | 840,208 | 14.2 |  | 841,933 | 14.2 |  | 681,413 | 11.5 | -19% |
| **Other** | 449,774 | 7.6 |  | 452,366 | 7.6 |  | 528,242 | 8.9 | +17% |
| **Unknown** | 1,498,009 | 25.3 |  | 1,496,761 | 25.2 |  | 1,308,191 | 22.1 | -13% |

**Supplementary Table 2. Sequence identity of full-length SILVA references used to generate synthetic read sets for Fig 2 and Fig S3-5 compared to the most similar reference sequence in the ‘Classifier’ database.** Synthetic read sets were constructed using 1,013 randomly selected bacterial 16S sequences from the SILVA^8^ (release 119) database (see methods). The same full-length SILVA references were compared to the ‘Classifier’ reference database (Greengenes^15^, 99% OTU clustering) using BLAST^16^ to determine sequence identity. Almost half of the SILVA reference sequences used have only imperfect matches in the ‘Classifier’ reference database. Only reference with ≥97% sequence identity were used to construct synthetic read sets for **Fig. 2**, **Supplementary Fig 5, 7 & 8**.

| **%ID**  **(SILVA vs. Greengenes)** | ***n*** | **%** |
| --- | --- | --- |
| 100 | 545 | 53.8 |
| 99.5-99.99 | 261 | 25.8 |
| 99-99.49 | 117 | 11.5 |
| 98.5-98.99 | 31 | 3.1 |
| 98-98.49 | 26 | 2.6 |
| 97.5-97.99 | 22 | 2.2 |
| 97-97.49 | 11 | 1.1 |
| **Total** | **1,013** | **100** |

**Supplementary Table 3. Broad taxonomic classification of read 1 versus read 2 by SURPI differs for 2-9% of mate pairs.** Broad taxonomic classification by SURPI^14^ (as per Fig 2d) was determined for read 1 and read 2 of paired synthetic reads (SILVA 119, see methods) and RNA-Seq data (samples from Fig 1b, limited to pairs passing quality filters, see methods). Broad taxonomic assignments were compared for concordance. Discordance ranged between 2-3% for synthetic 16S read pairs and from 3-9% for RNA-Seq data. Discordance was greatest for samples with higher abundance of bacterial reads (samples 2 and 3, Fig 1b), presumably due to database incompleteness, inconsistent annotations, and because SURPI’s assignment is based on the single reference sequence with the highest score.

| **Sample** | **Read Length** | **Read pairs with discordant assignment, R1 vs. R2 (*n*)** | **Total  pairs (*n*)** | **%** |
| --- | --- | --- | --- | --- |
| Synthetic 16S | 2x100bp | 6,984 | 300,128 | 2.3 |
| Synthetic 16S | 2x250bp | 2,888 | 119,009 | 2.4 |
| Sample 1 (Table 1) | 2x100bp | 172,759 | 5,916,921 | 2.9 |
| Sample 2 (Table 1) | 2x100bp | 586,486 | 6,261,301 | 9.4 |
| Sample 3 (Table 1) | 2x100bp | 326,263 | 5,536,276 | 5.9 |

**Supplementary Table 4.** Accessions for published 16S amplicon data used in for bacterial abundance estimates (**Fig. 2e**), numbers of reads, and analysis times for the RDP Classifier and Taxonomer. Number of reads for reference ^12^ is based on mate pairs.

| **Sample** | **Source** | **Ref.** | **Reads** | **RDP Classifier [min]** | **Taxonomer  [min]** |
| --- | --- | --- | --- | --- | --- |
| ERR498444 | Human gut | ^11^ | 20,469 | 285 | 0.91 |
| ERR498459 | Human gut | ^11^ | 8,413 | 303 | 0.45 |
| ERR498467 | Human gut | ^11^ | 16,864 | 426 | 0.62 |
| ERR498476 | Human gut | ^11^ | 19,066 | 402 | 0.96 |
| ERR498532 | Human gut | ^11^ | 20,458 | 315 | 1.02 |
| ERR498541 | Human gut | ^11^ | 18,803 | 354 | 0.78 |
| ERR498566 | Human gut | ^11^ | 12,612 | 200 | 0.60 |
| ERR498576 | Human gut | ^11^ | 10,070 | 258 | 0.49 |
| ERR498611 | Human gut | ^11^ | 19,506 | 342 | 0.96 |
| ERR498653 | Human gut | ^11^ | 14,311 | 225 | 0.61 |
| ERR502969 | Dog nose | ^12^ | 62,836 | 492 | 1.26 |
| ERR502989 | Human nose | ^12^ | 79,093 | 930 | 2.37 |
| ERR503004 | Kitchen floor | ^12^ | 74,061 | 594 | 2.00 |
| ERR503007 | Human hand | ^12^ | 67,144 | 615 | 1.41 |
| ERR503052 | Human nose | ^12^ | 54,569 | 468 | 0.87 |
| ERR503054 | Human hand | ^12^ | 77,382 | 822 | 2.67 |
| ERR503166 | Bedroom floor | ^12^ | 57,718 | 498 | 1.10 |
| ERR503209 | Kitchen floor | ^12^ | 64,996 | 534 | 1.91 |
| ERR503211 | Bathroom door knob | ^12^ | 70,964 | 630 | 1.44 |
| ERR503212 | Human nose | ^12^ | 124,363 | 852 | 3.27 |

**Supplementary Table 5.** Genes (n=17) that are differentially regulated in nasopharyngeal and oropharyngeal swabs from children with pneumonia who tested positive for influenza virus (n=4) compared to asymptomatic controls (n=40). Read counts and p-values (raw and adjusted) are shown. A – controls; B - influenza

| **Gene ID** | **Base Mean A** | **Base Mean B** | **Fold Change** | **p** | **p(adj)** |
| --- | --- | --- | --- | --- | --- |
| IFIT1 | 0.7 | 73.4 | 104.5 | 7.1E-19 | 1.5E-14 |
| IFI6 | 0.5 | 31.4 | 64.8 | 6.3E-13 | 6.7E-09 |
| IFIT2 | 2.1 | 135.5 | 63.8 | 7.8E-09 | 5.5E-05 |
| ISG15 | 1.4 | 61.2 | 43.3 | 1.4E-08 | 6.4E-05 |
| OASL | 0.6 | 20.3 | 33.3 | 1.5E-08 | 6.4E-05 |
| IFIT3 | 2.1 | 81.2 | 38.7 | 5.4E-08 | 1.9E-04 |
| NT5C3A | 0.7 | 20.1 | 30.7 | 3.3E-07 | 9.9E-04 |
| MX2 | 1.4 | 27.4 | 19.2 | 4.0E-07 | 1.1E-03 |
| IFITM1 | 2.4 | 32.8 | 14.0 | 6.4E-07 | 1.5E-03 |
| CXCL10 | 0.6 | 37.3 | 64.6 | 9.0E-07 | 1.9E-03 |
| IFI44L | 1.5 | 26.6 | 17.8 | 1.6E-06 | 3.1E-03 |
| MX1 | 4.2 | 56.5 | 13.5 | 1.8E-06 | 3.2E-03 |
| IFIH1 | 1.4 | 21.3 | 15.0 | 9.7E-06 | 1.6E-02 |
| OAS2 | 2.8 | 37.5 | 13.2 | 1.3E-05 | 1.9E-02 |
| SAMD9 | 2.8 | 61.9 | 22.5 | 2.6E-05 | 3.7E-02 |
| RSAD2 | 1.4 | 47.0 | 33.7 | 2.9E-05 | 3.8E-02 |
| DDX58 | 1.1 | 16.6 | 15.3 | 3.9E-05 | 4.8E-02 |

**Supplementary Table 6.** Taxonomer is compatible with different sequencing protocols. Two samples known to be positive for influenza A virus H1N1 and (nasopharyngeal swab) and *Mycoplasma pneumoniae* (bronchoalveolar lavage) based on diagnostic PCR test were analyzed by 3 commonly-used next-generation sequencers (illumina MiSeq, illumine HiSeq, Life Technologies Ion Proton). While the same sequencing libraries were analyzed with the MiSeq and HiSeq instruments, separate sequencing libraries were prepared for the Ion Proton instrument. Similar proportions of viral (0.43% to 0.55% of all reads) and bacterial (16S rRNA sequences representing 0.004% to 0.006% of all reads) pathogen sequences were obtained with all experimental conditions.

|  | **MiSeq (2 x 250 bp)** | | | | | |  | | **HiSeq (2 x 100 bp)** | | | | | |  | | **Ion Proton (1 x ~140 bp)** | | | | | |  |
| --- | --- | --- | --- | --- | --- | --- | --- | --- | --- | --- | --- | --- | --- | --- | --- | --- | --- | --- | --- | --- | --- | --- | --- |
| **Bin** | **Reads (*n*)** | | **%** | | **Pathogen reads (%)** | |  | | **Reads (*n*)** | | **%** | | **Pathogen reads (%)** | |  | | **Reads (*n*)** | | **%** | | **Pathogen reads (%)** | |  |
| **Influenza A** | | | | | | | | | | | | | | | | | | | | | | |  |
| Human | | 2,383,619 | | 78.3 | |  | |  | | 23,365,714 | | 80.8 | |  | |  | | 16,004,966 | | 86.0 | |  | |
| Fungal | | 2,824 | | 0.1 | |  | |  | | 57,141 | | 0.2 | |  | |  | | 88,237 | | 0.5 | |  | |
| ITS | | 101 | | 0.0 | |  | |  | | 3,065 | | 0.0 | |  | |  | | 1,088 | | 0.0 | |  | |
| Bacteria | | 105,307 | | 3.5 | |  | |  | | 922,407 | | 3.2 | |  | |  | | 783,684 | | 4.2 | |  | |
| 16S | | 21,776 | | 0.7 | |  | |  | | 215,163 | | 0.7 | |  | |  | | 501,958 | | 2.7 | |  | |
| Phage | | 1,328 | | 0.0 | |  | |  | | 2,943 | | 0.0 | |  | |  | | 3 | | 0.0 | |  | |
| **Viral** | | **44,670** | | **1.5** | | **99.5** | |  | | **538,290** | | **1.9** | | **99.3** | |  | | **467,567** | | **2.5** | | **99.2** | |
| Other | | 476,256 | | 15.7 | |  | |  | | 3,786,107 | | 13.1 | |  | |  | | 719,070 | | 3.9 | |  | |
| Ambiguous | |  | |  | |  | |  | |  | |  | |  | |  | |  | |  | |  | |
| Unknown | | 3,958 | | 0.1 | |  | |  | | 26,939 | | 0.1 | |  | |  | | 36,934 | | 0.2 | |  | |
| **Mycoplasma** | | | | | | | | | | | | | | | | | | | | | | |  |
| Human | 3,015,081 | | 88.2 | |  | |  | | 26,438,464 | | 89.9 | |  | |  | | 15,464,350 | | 94.4 | |  | |  |
| Fungal | 3,290 | | 0.1 | |  | |  | | 67,372 | | 0.2 | |  | |  | | 42,548 | | 0.3 | |  | |  |
| ITS | 48 | | 0.0 | |  | |  | | 3,636 | | 0.0 | |  | |  | | 459 | | 0.0 | |  | |  |
| Bacteria | 1,749 | | 0.1 | |  | |  | | 9,454 | | 0.0 | |  | |  | | 7,917 | | 0.1 | |  | |  |
| **16S** | **309** | | **0.0** | | **69.3** | |  | | **2,951** | | **0.0** | | **65.9** | |  | | **2,650** | | **0.0** | | **30.5** | |  |
| Phage | 1,270 | | 0.0 | |  | |  | | 3,158 | | 0.0 | |  | |  | | 0 | | 0.0 | |  | |  |
| Viral | 64 | | 0.0 | |  | |  | | 509 | | 0.0 | |  | |  | | 295 | | 0.0 | |  | |  |
| Other | 391,584 | | 11.5 | |  | |  | | 2,834,912 | | 5.0 | |  | |  | | 825,431 | | 5.0 | |  | |  |
| Ambiguous |  | |  | |  | |  | |  | |  | |  | |  | |  | |  | |  | |  |
| Unknown | 1,255 | | 0.0 | |  | |  | | 20,841 | | 0.1 | |  | |  | | 34,997 | | 0.2 | |  | |  |

**Supplementary Table 7.** Reference databases for the ‘Binner’ module, source, version, number of reference sequences, and k-mers.

| **Binner Database** | **Flag** | **Source** | **Version** | **Sequences** | **k-mers** | **Source** |
| --- | --- | --- | --- | --- | --- | --- |
| Human genome | 2 | NCBI SILVA | GRCh38^1^ 4/10/2014 | 455  +8 LSU  + 158 SSU | 2,257,262,659 | [ftp.ncbi.nlm.nih.gov/genomes/Homo_sapiens/Assembled_chromosomes/seq/](ftp://ftp.ncbi.nlm.nih.gov/genomes/Homo_sapiens/Assembled_chromosomes/seq/)  <http://www.arb-silva.de/documentation/release-119/> |
| Human transcripts | 32 | NCBI | 4/10/2014 | 98,746 | 79,809,821 | <ftp://ftp.ncbi.nlm.nih.gov/genomes/H_sapiens/RNA/> |
| Human mitochondria | 512 | Mitomap | 4/10/2014 | 26,829 | 248,056 | <http://mitomap.org/bin/view.pl/MITOMAP/Mitobank> |
| Bacterial genomes | 1 | NCBI | 4/10/2014 | 5,189 | 5,433,934,380 | [ftp.ncbi.nlm.nih.gov/genomes/](ftp://ftp.ncbi.nlm.nih.gov/genomes/) |
| Bacterial SSU | 128 | SILVA | 119_ref | 1,473,625 | 45,354,161 | <http://www.arb-silva.de/documentation/release-119/> |
| Bacterial LSU | 256 | SILVA | 119_ref | 41,505 | 4,817,477 | <http://www.arb-silva.de/documentation/release-119/> |
| Plastids LSU/SSU | 16384 | SILVA | 119_ref | 2,946 LSU  10,458 SSU | 2,148,466 | <http://www.arb-silva.de/documentation/release-119/> |
| Fungal genomes | 4 | NCBI | 4/10/2014 | 1,146 | 583,739,768 | [ftp.ncbi.nlm.nih.gov/genomes/](ftp://ftp.ncbi.nlm.nih.gov/genomes/) |
| Fungal LSU/SSU | 64 | SILVA | 119_ref | 2,317 LSU  21,106 SSU | 2,306,754 | <http://www.arb-silva.de/documentation/release-119/> |
| Fungal ITS | 1024 | UNITE | 07/04/2014 | 409,493 | 19,398,519 | <https://unite.ut.ee/index.php> |
| Viruses (NCBI) | 8 | NCBI | 8/ 7/2014 | 1,668,565 | 279,444,799 | entrez search with query (txid10239[Organism] and not txid131567[Organism] and not gbdiv_pat[PROP]) |
| Phage | 16 | NCBI | 4/10/2014 | 14,440 | 69,802,836 | Phage taxID’s per viralzone: <http://viralzone.expasy.org/all_by_protein/256.html> and <http://viralzone.expasy.org/all_by_species/663.html> |
| Phi X174 | 4096 | NCBI |  | 1 | 5,366 | NC_001422 |
| Other Eukaryotes LSU/SSU | 2048 | SILVA | 119_ref | 86,633 | 16,062,966 | <http://www.arb-silva.de/documentation/release-119/> |
| Repeat masker | 8192 | Repeatmasker |  | 383 | 16,419 | <http://www.repeatmasker.org/> |
| Adapter sequences |  |  |  |  |  |  |
| ERCC controls |  |  |  |  |  |  |

SSU – small subunit rRNA

LSU – large subunit rRNA

ITS – internal transcribed spacer

**Supplementary Table 8.** **Bin assignment for reads with equal numbers of k-mer matches to multiple Binner databases and k-mer matches below threshold.** Some reference sequence databases are subsets or overlap with others (e.g. ‘Human transcripts’ and ‘Human genome’) and some sequences may be assigned varying taxID’s (e.g. phage sequences may be annotated as viruses or as bacteria, if integrated as prophages). As a result, query sequences may share an equal number of k-mers with more than one reference database. The ‘Binner’ module assigns these query sequences as outlined below.

| **Equal k-mer count of…** | **And…** | **Assignment** |
| --- | --- | --- |
| ‘Human transcripts’ | ‘Human genome’ and/or ‘Mitochondrial genomes’ | ‘Human transcripts’ |
| ‘Bacterial 16S’ | ‘Bacterial LSU’ and/or ‘Bacterial genomes’ and/or ‘Plastids LSU/SSU’ | ‘Bacterial 16S’ |
| ‘Fungal ITS’ | ‘Fungal genomes’ and/or ‘Fungal LSU/SSU’ | ‘Fungal ITS’ |
| ‘Phage’ | ‘Viruses (NCBI)’ and/or ‘Bacterial genomes’ | ‘Phage’ |
| All other ties |  | ‘Ambiguous’ |
| K-mer count < threshold |  | ‘Unknown’ |

**Supplementary Table 9. Contents of visualized pie charts in the web portal.** Sub-bin assignments are summarized for interactive visualization at taxonomer.iobio.io as indicated.

| **Bin** | **Sub-bins** |
| --- | --- |
| Human | ‘Human genome’, ‘Human transcripts’, ‘Mitochondrial genomes’ |
| Bacterial | ‘Bacterial genomes’, ‘Bacterial SSU’, ‘Bacterial LSU’, ‘Plastids LSU/SSU’ |
| Fungal | ‘Fungal genomes’, ‘Fungal LSU/SSU’, ‘Fungal ITS’ |
| Viral | ‘Viruses (NCBI)’, ‘Phage’ |
| Other | ‘Other Eukaryotes LSU/SSU’ |
| Ambiguous | Any database combination not specified above |

**Supplementary Table 10. Optimal k-mer cutoffs for bin assignments based on the Youden’s Index and F1 Score.** Optimal k-mer cutoffs determined by receiver operator characteristics analysis (**Supplementary Fig. 1b**) using the Youden’s Index and F1 Score^1^ are shown. The default cutoff used by the ‘Binner’ module is 11.

|  | **Youden’s Index** | **F1 Score** |
| --- | --- | --- |
| Human | 13 | 13 |
| Bacteria | 5 | 8 |
| Fungal | 3 | 4 |
| Virus | 3 | 4 |
| Parasite | 22 | 21 |

* Parasites are not present in the binner databases, reads from parasites are considered true positives if they remain unbinned

**Supplementary Table 11.** Viruses, percent nucleotide-level identity to reference sequences in the NCBI nt database, as well as numbers of total and viral reads for pediatric upper respiratory tract specimens used to compare ‘Protonomer’, RAPSearch2, and DIAMOND for protein-level classification of viral sequences (**Fig. 3**, **Supplementary Fig. 3 & 4**). HCoV – human coronavirus, HBoV – human bocavirus, HMPV – human metapneumovirus, HRV – rhinovirus, PIV – parainfluenza virus, RSV – respiratory syncytial virus.

| **Virus** | **Sample Accession** | **Nucleotide ID** | **GenBank Accession** | **Total Reads** | **Target Reads (*n*)** | **Target Reads (%)** |
| --- | --- | --- | --- | --- | --- | --- |
| HCoV (HKU1) | ERS1118338 | 99.8% | KF686344 | 317,354 | 305,544 | 96.3% |
| HCoV (NL63) | ERS1118339 | 99.8% | JQ765567 | 44,825 | 20,800 | 46.4% |
| HCoV (OC43) | ERS1118340 | 99.7% | AY903460 | 15,515 | 6,919 | 44.6% |
| Coxsackie B4 | ERS1118341 | 84.1% | KF878966 | 21,399 | 1,027 | 4.8% |
| HBoV | ERS1118342 | 99.6% | JQ923422 | 206,869 | 1,119 | 0.5% |
| HMPV | ERS1118343 | 98.5% | GQ153651 | 80,362 | 7,059 | 8.8% |
| HMPV | ERS1118347 | 99.0% | EF535506 | 55,240 | 2,683 | 4.9% |
| HRV-A | ERS1118350 | 90.9% | EF173415 | 11,369 | 2,413 | 21.2% |
| HRV-C | ERS1118344 | 85.2% | DQ875932.2 | 490,829 | 491 | 0.10% |
| HRV-C | ERS1118345 | 85.3% | DQ875932.2 | 704,819 | 394 | 0.06% |
| HRV-C | ERS1118346 | 79.3% | JF436925.1 | 662,784 | 200 | 0.03% |
| HRV-C | ERS1118351 | 97.3% | JX074056 | 385,808 | 208,446 | 54.0% |
| HRV-C | ERS1118352 | 82.1% | JF317017 | 306,436 | 232,451 | 75.9% |
| HRV-C | ERS1118353 | 97.2% | JX074056 | 246,973 | 35,474 | 14.4% |
| HRV-C | ERS1118354 | 75.9% | KF958311 | 28,862 | 2,657 | 9.2% |
| HRV-C | ERS1118355 | 96.0% | JN990702 | 330,157 | 252,416 | 76.5% |
| HRV-C | ERS1118356 | 76.5% | GQ223228 | 179,888 | 153,429 | 85.3% |
| HRV-C | ERS1118357 | 95.4% | GQ323774 | 58,005 | 1,369 | 2.4% |
| PIV-1 | ERS1118348 | 99.2% | JQ901989 | 107,818 | 9,392 | 8.7% |
| PIV-3 | ERS1118349 | 99.4% | KF530232 | 48,547 | 15,651 | 32.2% |
| RSV-A | ERS1118359 | 99.7% | KF826849.1 | 762,085 | 2,218 | 0.29% |
| RSV-B | ERS1118358 | 97.9% | JQ582843 | 1,784 | 1,035 | 58.0% |
| RSV-B | ERS1118360 | 97.9% | JQ582843 | 40,707 | 32,047 | 78.7% |
| RSV-B | ERS1118361 | 99.7% | JN032120.1 | 516,693 | 495,469 | 95.9% |

**Supplementary Table 12.** Ascension numbers for human brain RNAseq data used to compare with MAQC qPCR data^17^.

| **Sample** | **Source** | **Reads** |
| --- | --- | --- |
| SRR037452 | Human brain | 11,712,885 |
| SRR037453 | Human brain | 11,413,794 |
| SRR037454 | Human brain | 11,816,021 |
| SRR037455 | Human brain | 11,244,980 |
| SRR037456 | Human brain | 12,081,324 |
| SRR037457 | Human brain | 11,365,146 |
| SRR037458 | Human brain | 11,616,331 |

**Supplementary Table 13.** Flux Simulator^18^ parameters used to generate simulated RNAseq reads for benchmarking transcript assignment. Following the benchmarks used for Sailfish we filtered the transcript GTF using the gffread utility with the flags -C -M -E and -T, as well as any transcripts consisting soley of Ns. The GTF was sorted using the FluxSimulator sortGTF command and used to generate the synthetic data for bencmarking.

| **Stage** | **Parameters** |
| --- | --- |
| Expression | NB_MOLECULES 5000000  REF_FILE_NAME Homo_sapiens_ENSMBL_37.75.gtf  TSS_MEAN 50  POLYA_SCALE NaN  POLYA_SHAPE NaN |
| Fragmentation | FRAG_SUBSTRATE RNA  FRAG_METHOD UR  FRAG_UR_ETA NaN  FRAG_UR_D0 1 |
| Reverse Transcription | RTRANSCRIPTION YES  RT_PRIMER RH  RT_LOSSLESS YES  RT_MIN 500  RT_MAX 5500 |
| Filtering & Amplification | FILTERING YES  GC_MEAN NaN  PCR_PROBABILITY 0.05 |
| Sequencing | READ_NUMBER 150000000  READ_LENGTH 76  PAIRED_END YES  ERR_FILE 76  FASTA YES  UNIQUE_IDS NO |

**REFERENCES**

1. Akobeng, A.K. Understanding diagnostic tests 3: Receiver operating characteristic curves. *Acta Paediatr* **96**, 644-647 (2007).

2. Rinke, C. et al. Insights into the phylogeny and coding potential of microbial dark matter. *Nature* **499**, 431-437 (2013).

3. Nielsen, H.B. et al. Identification and assembly of genomes and genetic elements in complex metagenomic samples without using reference genomes. *Nature biotechnology* (2014).

4. Jain, S. et al. Incidence and Etiology of Community-Acquired Pneumonia Requiring Hospitalization among U.S. Children. *The New England journal of medicine*, In press (2014).

5. Zhao, Y., Tang, H. & Ye, Y. RAPSearch2: a fast and memory-efficient protein similarity search tool for next-generation sequencing data. *Bioinformatics* **28**, 125-126 (2012).

6. Buchfink, B., Xie, C. & Huson, D.H. Fast and sensitive protein alignment using DIAMOND. *Nature methods* (2014).

7. Li, H. & Durbin, R. Fast and accurate short read alignment with Burrows-Wheeler transform. *Bioinformatics* **25**, 1754-1760 (2009).

8. Yilmaz, P. et al. The SILVA and "All-species Living Tree Project (LTP)" taxonomic frameworks. *Nucleic acids research* **42**, D643-648 (2014).

9. Cole, J.R. et al. Ribosomal Database Project: data and tools for high throughput rRNA analysis. *Nucleic acids research* **42**, D633-642 (2014).

10. Wood, D.E. & Salzberg, S.L. Kraken: ultrafast metagenomic sequence classification using exact alignments. *Genome biology* **15**, R46 (2014).

11. Subramanian, S. et al. Persistent gut microbiota immaturity in malnourished Bangladeshi children. *Nature* **510**, 417-421 (2014).

12. Lax, S. et al. Longitudinal analysis of microbial interaction between humans and the indoor environment. *Science* **345**, 1048-1052 (2014).

13. Gire, S.K. et al. Genomic surveillance elucidates Ebola virus origin and transmission during the 2014 outbreak. *Science* **345**, 1369-1372 (2014).

14. Naccache, S.N. et al. A cloud-compatible bioinformatics pipeline for ultrarapid pathogen identification from next-generation sequencing of clinical samples. *Genome research* **24**, 1180-1192 (2014).

15. DeSantis, T.Z. et al. Greengenes, a chimera-checked 16S rRNA gene database and workbench compatible with ARB. *Applied and environmental microbiology* **72**, 5069-5072 (2006).

16. Altschul, S.F., Gish, W., Miller, W., Myers, E.W. & Lipman, D.J. Basic local alignment search tool. *Journal of molecular biology* **215**, 403-410 (1990).

17. Bullard, J.H., Purdom, E., Hansen, K.D. & Dudoit, S. Evaluation of statistical methods for normalization and differential expression in mRNA-Seq experiments. *BMC bioinformatics* **11**, 94 (2010).

18. Griebel, T. et al. Modelling and simulating generic RNA-Seq experiments with the flux simulator. *Nucleic acids research* **40**, 10073-10083 (2012).
